# Correction: A randomised double blind placebo controlled phase 2 trial of adjunctive aspirin for tuberculous meningitis in HIV-uninfected adults

**DOI:** 10.7554/eLife.87888

**Published:** 2023-03-21

**Authors:** Nguyen Thi Hoang Mai, Nicholas Dobbs, Nguyen Hoan Phu, Romain A Colas, Le TP Thao, Nguyen TT Thuong, Ho DT Nghia, Nguyen HH Hanh, Nguyen T Hang, A Dorothee Heemskerk, Jeremy N Day, Lucy Ly, Do DA Thu, Laura Merson, Evelyne Kestelyn, Marcel Wolbers, Ronald Geskus, David Summers, Nguyen VV Chau, Jesmond Dalli, Guy E Thwaites

**Keywords:** Human

Mai NTH, Dobbs N, Phu NH, Colas RA, Thao LTP, Thuong NTT, Nghia HDT, Hanh NHH, Hang NT, Heemskerk AD, Day JN, Ly L, Thu DDA, Merson L, Kestelyn E, Wolbers M, Geskus R, Summers D, Chau NVV, Dalli J, Thwaites GE. 2018. A randomised double blind placebo controlled phase 2 trial of adjunctive aspirin for tuberculous meningitis in HIV-uninfected adults. *eLife* **7**:e33478. doi: 10.7554/eLife.33478.

Published 27 February 2018

We have recently been notified via PubPeer that the illustration we employed in our publications to denote presence of lipid mediators in the samples of interest has been interpreted as raw data. As this panel was strictly meant to be an illustration, we provide a revised figure presenting chromatograms supporting the presence of each of the reported mediators.

The corrected Figure 3—figure supplement 1 is shown here:

**Figure 1:**
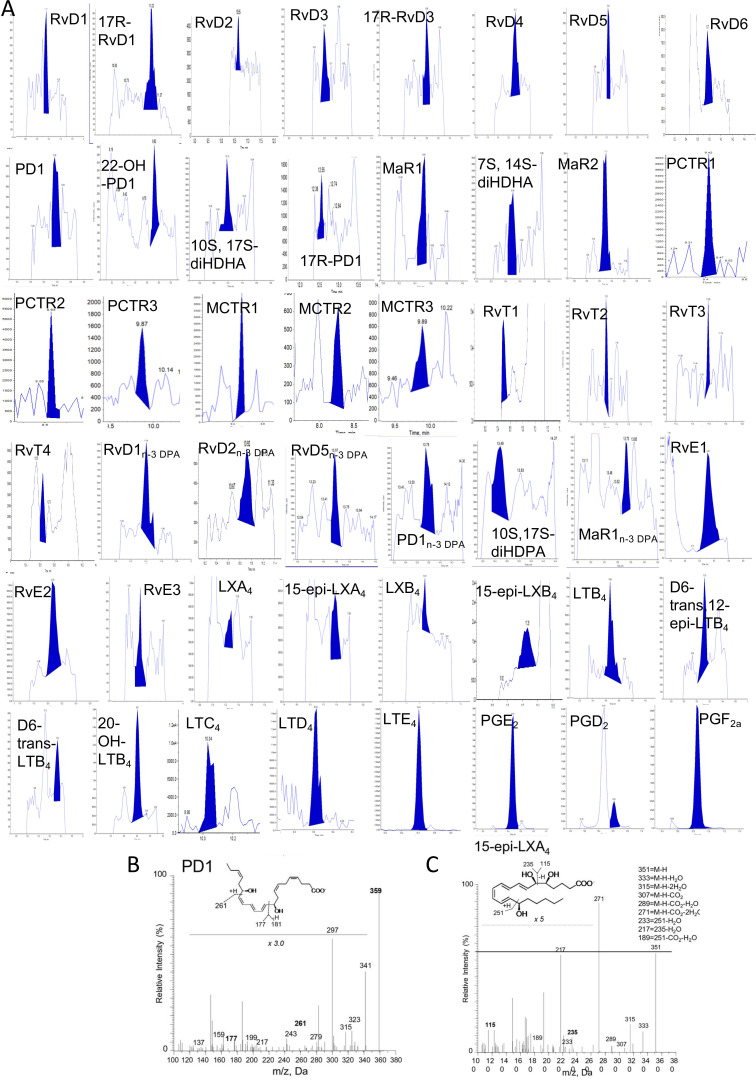


The original Figure 3—figure supplement 1 is shown here for reference:

**Figure 2:**
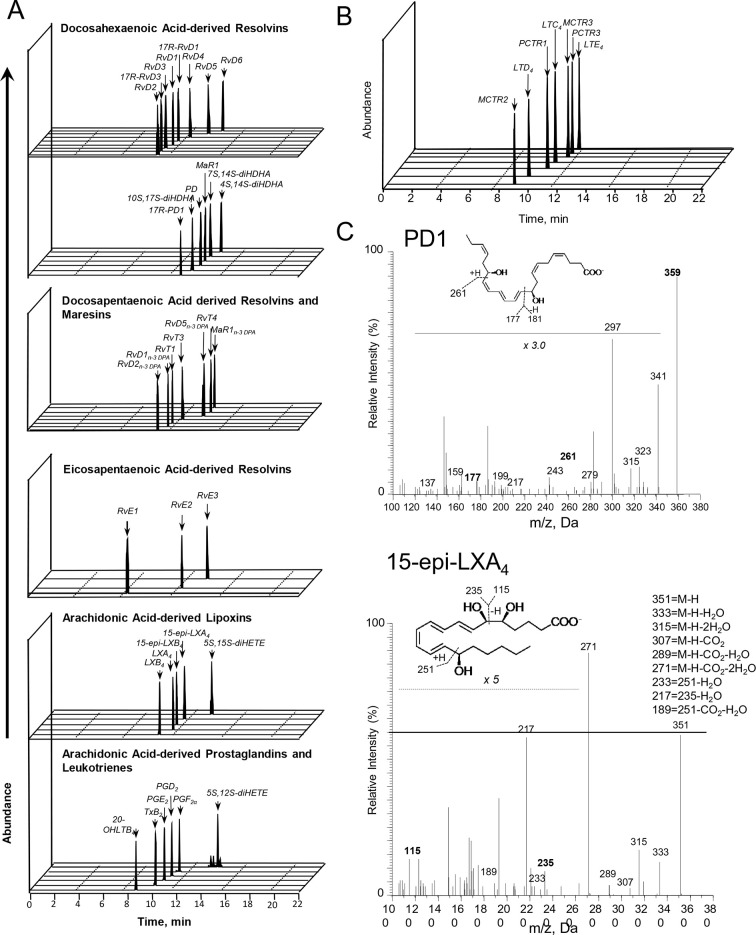


We have also clarified the Data availability statement. The revised statement reads:

The Oxford University Clinical Research Unit (OUCRU) operates managed open access to the research data it generates, which complies with the policies of its major funder, the Wellcome Trust, UK. The objective is not to restrict access to data, but to monitor who uses the data and for what purpose, and to ensure those responsible for collecting and curating the data are appropriately acknowledged by those using it. Therefore, those wishing to acquire the anonymized dataset, including LC-MS/MS data, from which the results presented in this manuscript were produced should email the trial Chief Investigator and corresponding author, Professor Guy Thwaites (gthwaites@oucru.org).

## The original statement read

The Oxford University Clinical Research Unit (OUCRU) operates managed open access to the research data it generates, which complies with the policies of its major funder, the Wellcome Trust, UK. The objective is not to restrict access to data, but to monitor who uses the data and for what purpose, and to ensure those responsible for collecting and curating the data are appropriately acknowledged by those using it. Therefore, those wishing to acquire the anonymized dataset from which the results presented in this manuscript were produced should email the trial Chief Investigator and corresponding author, Professor Guy Thwaites (gthwaites@oucru.org).

The article has been corrected accordingly.

