# Supplementary material for: A randomised double blind placebo controlled phase 2 trial of adjunctive aspirin for tuberculous meningitis in HIV-uninfected adults
Source: eLife. 2018 Feb 27;7:e33478. doi: 10.7554/eLife.33478 (PMC5862527; doi:10.7554/eLife.33478)
Supplement: Supplementary file 2. [file elife-33478-supp2.docx]

**Table S2. Reasons for exclusion of screened patients from the trial**

| **Exclusion criteria met** | **Number of subjects** |
| --- | --- |
| HIV infection | 10 |
| Unlikely, for any reason, to be able to have an MRI brain scan within 5 days (120 hours) of randomisation | 3 |
| Known or suspected infection with multi-drug resistant tuberculosis (resistant to at least isoniazid and rifampicin) | 2 |
| Unable to take isoniazid, rifampicin, or pyrazinamide at recommended doses for any reason | 2 |
| History of diagnosed peptic ulceration or gastro-intestinal bleeding | 1 |
| Active gastro-intestinal bleeding is suspected | 2 |
| Aspirin considered to be contraindicated for any reason by the attending physician | 1 |
| Pregnancy or breast feeding (negative urine pregnancy test for all females of child-bearing age) | 4 |
| Any other significant disease or disorder which, in the opinion of the Investigator, may either put the participants at risk because of participation in the study, or may influence the result of the study, or the participant’s ability to participate in the study. | 5 |
| Inform consent not given | 10 |
| Other diagnosis other than TBM | 33 |
