# Supplementary material for: A randomised double blind placebo controlled phase 2 trial of adjunctive aspirin for tuberculous meningitis in HIV-uninfected adults
Source: eLife. 2018 Feb 27;7:e33478. doi: 10.7554/eLife.33478 (PMC5862527; doi:10.7554/eLife.33478)
Supplement: Supplementary file 3. [file elife-33478-supp3.docx]

**Table S3. Summary of adverse events related, or possibly related, to aspirin**

| **Adverse event** | **Placebo (n=41)**  No. patients (%) (number of events) | **Aspirin 81mg (n=39)**  No. patients (%) (number of events) | **Aspirin 1000mg (n=40)**  No. patients (%) (number of events) | **Comparison P-value** |
| --- | --- | --- | --- | --- |
| **Any selected adverse event** | **7 (17.1%) (7)** | **9 (23.1%) (12)** | **7 (17.5%) (7)** | **0.81** |
| **ALLERGIC EVENTS** | **1 (2.4%) (1)** | **2 (5.1%) (4)** | **0 (0%) (0)** | **0.32** |
| - Urticaria/rash | 1 (2.4%) (1) | 2 (5.1%) (2) | 0 (0%) (0) | 0.32 |
| - Stevens Johnsons syndrome† | 0 (0%) | 1 (2.6%) (2) | 0 (0%) (0) | 0.33 |
| **GASTROINTESTINAL EVENTS** | **5 (12.2%) (5)** | **7 (17.9%) (8)** | **7 (17.5%) (7)** | **0.78** |
| - Haematemesis (changed or frank blood any volume) | 2 (4.9%) (2) | 1 (2.6%) | 2 (5%) (2) | 1.00 |
| - Greater than 5mls of fresh or changed blood aspirated from naso-gastric tube | 2 (4.9%) (2) | 6 (15.4%) (6) | 4 (10%) (4) | 0.26 |
| - Melena (black tarry stool) | 1 (2.4%) (1) | 1 (2.6%) (1) | 1 (2.5%) (1) | 1.00 |
| **OTHER ADVERSE EVENTS** | **1 (2.4%) (1)** | **0 (0%) (0)** | **0 (0%) (0)** | **1.00** |
| - metabolic acidosis | 1 (2.4%) (1) | 0 (0%) (0) | 0 (0%) (0) | 1.00 |

† Considered more likely to be associated with rifampicin, but aspirin link could not be excluded.
