# Supplementary material for: A randomised double blind placebo controlled phase 2 trial of adjunctive aspirin for tuberculous meningitis in HIV-uninfected adults
Source: eLife. 2018 Feb 27;7:e33478. doi: 10.7554/eLife.33478 (PMC5862527; doi:10.7554/eLife.33478)
Supplement: Supplementary file 4. [file elife-33478-supp4.docx]

**Table S4. Full Rankin scores by treatment group by day 60 and 8 months in the ITT population**

|  | Placebo (N=41) | Aspirin_81mg (N=39) | Aspirin_1000mg (N=40) | P-value |
| --- | --- | --- | --- | --- |
| **Rankin Scores by 60 days †** |  |  |  |  |
| - No symptoms | 6/38(15.8%) | 11/39(28.2%) | 9/40(22.5%) | 0.40 |
| - Mild symptoms; no significant disability | 6/38(15.8%) | 9/39(23.1%) | 13/40(32.5%) |  |
| - Slight disability; but self-caring | 17/38(44.7%) | 7/39(18.0%) | 9/40(22.5%) |  |
| - Moderate disability; able to walk without assistance | 4/38(10.5%) | 3/39(7.7%) | 4/40(10.0%) |  |
| - Moderately severe; unable to walk without assistance | 3/38(7.9%) | 1/39(2.6%) | 2/40(5.0%) |  |
| - Severe disability; bed-ridden, requiring constant care | 0/38(0%) | 2/39(5.1%) | 2/40(5.0%) |  |
| - Death | 2/38(5.3%) | 6/39(15.4%) | 1/40(2.5%) |  |
| **Rankin Scores by 8 months** |  |  |  |  |
| - No symptoms | 19/39 (48.7%) | 23/39(59.0%) | 22/40(55.0%) | 0.25 |
| - Mild symptoms; no significant disability | 5/39 (12.8%) | 4/39(10.3%) | 7/40(17.5%) |  |
| - Slight disability; but self-caring | 5/39 (12.8%) | 3/39(7.7%) | 4/40(10.0%) |  |
| - Moderate disability; able to walk without assistance | 6/39(15.4%) | 2/39(5.1%) | 4/40(10.0%) |  |
| - Moderately severe; unable to walk without assistance | 1/39 (2.5%) | 0/39(0%) | 0/40(0%) |  |
| - Severe disability; bed-ridden, requiring constant care | 0/39 (0%) | 1/39(2.6%) | 0/40(0%) |  |
| - Death | 5/39 (12.8%) | 6/39(15.4%) | 3/40(7.5%) |  |

† 3 patients in the placebo group missed their day 60 assessment

 P-values refer to a linear-by-linear trend test for Rankin scores
