# Supplementary material for: A randomised double blind placebo controlled phase 2 trial of adjunctive aspirin for tuberculous meningitis in HIV-uninfected adults
Source: eLife. 2018 Feb 27;7:e33478. doi: 10.7554/eLife.33478 (PMC5862527; doi:10.7554/eLife.33478)
Supplement: Supplementary file 5. [file elife-33478-supp5.docx]

**Table S5. Other MRI brain findings by treatment group on days 60 and month 8 in the ITT population**

|  | **Placebo (N=41)** | **Aspirin 81mg (N=39)** | **Aspirin 1000mg (N=40)** | **Comparison**  **P-value** |
| --- | --- | --- | --- | --- |
| **Day 60 MRI brain imaging** |  |  |  |  |
| Tuberculomas seen | 16/35(45.7%) | 8/32(25.0%) | 14/38(36.8%) | 0.22 |
| Hydrocephalus | 8/35(22.9%) | 5/32(15.6%) | 2/38(5.3%) | 0.09 |
| Meningeal enhancement | 4/35(11.4%) | 4/32(12.5%) | 5/38(13.2%) | 1.00 |
| **Month 8 MRI brain imaging** |  |  |  |  |
| Infarcts seen | 10/33(30.3%) | 6/32(18.8%) | 10/37(27.0%) | 0.58 |
| Tuberculomas seen | 5/33(15.2%) | 3/32(9.4%) | 5/37(13.5%) | 0.81 |
| Hydrocephalus | 5/33(15.2%) | 2/32(6.3%) | 1/37(2.7%) | 0.16 |
| Meningeal enhancement | 0 | 0 | 0 | NA |
