# Supplementary material for: A randomised double blind placebo controlled phase 2 trial of adjunctive aspirin for tuberculous meningitis in HIV-uninfected adults
Source: eLife. 2018 Feb 27;7:e33478. doi: 10.7554/eLife.33478 (PMC5862527; doi:10.7554/eLife.33478)
Supplement: Supplementary file 6. [file elife-33478-supp6.docx]

| **DHA bioactive metabolome**  **Table S6. CSF individual lipid mediator Profiles at baseline and 30 days post aspirin and placebo administration†** | **Q1** | **Q3** | **Day 0 Placebo** | | | **Day 0 81mg** | | | **Day 0 1000mg** | | | **Day 30 Placebo** | | | **Day 30 81mg** | | | **Day 30 1000mg** | | |
| --- | --- | --- | --- | --- | --- | --- | --- | --- | --- | --- | --- | --- | --- | --- | --- | --- | --- | --- | --- | --- |
| RvD1 | 375 | 141 | 0.2 | ± | 0.0 | 0.1 | ± | 0.0 | 0.2 | ± | 0.1 | 0.4 | ± | 0.1 | 0.3 | ± | 0.0 | 0.2 | ± | 0.0 * |
| RvD2 | 375 | 141 | 0.5 | ± | 0.2 | 0.5 | ± | 0.2 | 0.6 | ± | 0.2 | 2.8 | ± | 0.7 | 2.6 | ± | 0.5 | 2.3 | ± | 0.5 |
| RvD3 | 375 | 147 |  | - |  | 0.1 | ± | 0.0 | 0.1 | ± | 0.0 | 0.1 | ± | 0.0 | 0.1 | ± | 0.0 | 0.1 | ± | 0.0 |
| RvD4 | 375 | 101 | 0.5 | ± | 0.1 | 0.7 | ± | 0.1 | 0.7 | ± | 0.1 | 0.3 | ± | 0.1 | 0.9 | ± | 0.5 | 0.3 | ± | 0.1 |
| RvD5 | 359 | 199 | 1.4 | ± | 0.3 | 1.8 | ± | 0.5 | 1.8 | ± | 0.4 | 0.2 | ± | 0.0 | 0.5 | ± | 0.3 | 0.3 | ± | 0.1 |
| RvD6 | 359 | 101 | 0.2 | ± | 0.1 | 0.5 | ± | 0.2 | 0.2 | ± | 0.1 | 0.2 | ± | 0.0 | 0.5 | ± | 0.3 | 0.2 | ± | 0.0 |
| 17R-RvD1 | 375 | 141 | 0.2 | ± | 0.0 | 0.2 | ± | 0.0 | 0.2 | ± | 0.0 | 0.2 | ± | 0.1 | 0.1 | ± | 0.0 | 0.1 | ± | 0.0 |
| 17R-RvD3 | 375 | 147 | 0.1 | ± | 0.0 | 0.1 | ± | 0.0 | 0.1 | ± | 0.0 |  | - |  | 0.1 | ± | 0.0 |  | - |  |
|  |  |  |  |  |  |  |  |  |  |  |  |  |  |  |  |  |  |  |  |  |
| PD1 | 359 | 153 | 0.6 | ± | 0.1 | 0.5 | ± | 0.1 | 0.8 | ± | 0.1 | 0.4 | ± | 0.1 | 0.5 | ± | 0.1 | 0.5 | ± | 0.1 |
| 10S,17S-diHDHA | 359 | 153 | 0.9 | ± | 0.1 | 1.1 | ± | 0.3 | 0.9 | ± | 0.3 | 0.2 | ± | 0.1 | 0.9 | ± | 0.6 | 0.4 | ± | 0.1 |
| 17R-PD1 | 359 | 153 |  | - |  | 0.1 | ± | 0.0 | 0.1 | ± | 0.0 |  | - |  | 0.1 | ± | 0.0 | 0.1 | ± | 0.0 |
| 22-OH-PD1 | 375 | 153 | 0.2 | ± | 0.1 | 0.2 | ± | 0.1 | 0.2 | ± | 0.1 | 0.6 | ± | 0.1 | 1.2 | ± | 0.5 | 0.8 | ± | 0.1 |
|  |  |  |  |  |  |  |  |  |  |  |  |  |  |  |  |  |  |  |  |  |
| PCTR1 | 650 | 231 | 0.5 | ± | 0.2 | 0.3 | ± | 0.1 | 0.1 | ± | 0.1 | 0.4 | ± | 0.3 | 0.4 | ± | 0.3 | 0.5 | ± | 0.4 |
| PCTR2 | 521 | 231 |  | - |  |  | - |  | 0.1 | ± | 0.1 | 0.1 | ± | 0.1 |  | - |  | 0.2 | ± | 0.2 |
| PCTR3 | 464 | 231 | 4.4 | ± | 1.8 | 2.5 | ± | 1.3 | 0.9 | ± | 0.5 | 2.8 | ± | 2.3 | 3.0 | ± | 1.7 | 2.1 | ± | 1.0 |
|  |  |  |  |  |  |  |  |  |  |  |  |  |  |  |  |  |  |  |  |  |
| MaR1 | 359 | 221 | 0.8 | ± | 0.2 | 0.5 | ± | 0.1 | 0.4 | ± | 0.1 | 0.5 | ± | 0.2 | 0.3 | ± | 0.1 | 0.6 | ± | 0.2 |
| 7S,14S-diHDHA | 359 | 221 | 1.9 | ± | 0.6 | 1.0 | ± | 0.3 | 2.4 | ± | 0.8 | 0.5 | ± | 0.2 | 2.7 | ± | 1.7 | 0.8 | ± | 0.5 |
| MaR2 | 359 |  | 24.6 | ± | 5.6 | 18.5 | ± | 4.8 | 21.4 | ± | 5.6 | 13.7 | ± | 3.6 | 7.1 | ± | 2.1 | 14.3 | ± | 3.6 |
| 22-OH-MaR1 | 375 | 221 | 0.3 | ± | 0.1 | 0.3 | ± | 0.1 | 0.4 | ± | 0.1 | 0.1 | ± | 0.0 |  | - |  | 0.1 | ± | 0.0 |
| 4S,14S-diHDHA | 359 | 101 | 0.4 | ± | 0.1 | 0.7 | ± | 0.2 | 0.9 | ± | 0.2 | 1.2 | ± | 0.3 | 1.1 | ± | 0.2 | 1.2 | ± | 0.2 |
|  |  |  |  |  |  |  |  |  |  |  |  |  |  |  |  |  |  |  |  |  |
| MCTR1 | 650 | 191 | 0.3 | ± | 0.2 | 0.1 | ± | 0.0 | 0.2 | ± | 0.1 |  | - |  | 0.1 | ± | 0.1 | 0.5 | ± | 0.2 * |
| MCTR2 | 521 | 191 |  | - |  | 0.2 | ± | 0.2 | 0.7 | ± | 0.5 | 0.2 | ± | 0.2 |  | - |  | 0.4 | ± | 0.2 |
| MCTR3 | 464 | 191 | 0.1 | ± | 0.1 | 0.2 | ± | 0.1 | 0.2 | ± | 0.1 | 1.8 | ± | 1.4 | 0.6 | ± | 0.4 | 0.2 | ± | 0.1 |
|  |  |  |  |  |  |  |  |  |  |  |  |  |  |  |  |  |  |  |  |  |
| **n-3 DPA bioactive metabolome** |  |  |  |  |  |  |  |  |  |  |  |  |  |  |  |  |  |  |  |  |
| RvT1 | 377 | 239 | 0.4 | ± | 0.1 | 0.5 | ± | 0.2 | 0.5 | ± | 0.1 | 0.2 | ± | 0.0 | 0.3 | ± | 0.1 | 0.2 | ± | 0.0 |
| RvT2 | 377 | 197 | 1.1 | ± | 0.2 | 1.1 | ± | 0.2 | 1.9 | ± | 0.4 | 1.5 | ± | 0.4 | 0.8 | ± | 0.2 | 1.2 | ± | 0.3 |
| RvT3 | 377 | 197 | 0.2 | ± | 0.1 | 0.1 | ± | 0.0 | 0.1 | ± | 0.0 | 0.1 | ± | 0.0 | 0.1 | ± | 0.0 | 0.1 | ± | 0.0 |
| RvT4 | 361 | 211 | 0.4 | ± | 0.1 | 0.3 | ± | 0.1 | 0.5 | ± | 0.1 | 0.1 | ± | 0.0 | 0.1 | ± | 0.0 | 0.2 | ± | 0.0 |
|  |  |  |  |  |  |  |  |  |  |  |  |  |  |  |  |  |  |  |  |  |
| RvD1_n-3 DPA_ | 377 | 143 | 0.5 | ± | 0.1 | 0.5 | ± | 0.2 | 0.4 | ± | 0.1 | 0.2 | ± | 0.0 | 0.2 | ± | 0.0 | 0.1 | ± | 0.0 |
| RvD2_n-3 DPA_ | 377 | 143 | 1.1 | ± | 0.2 | 1.0 | ± | 0.2 | 1.2 | ± | 0.2 | 0.2 | ± | 0.1 | 0.2 | ± | 0.0 | 0.2 | ± | 0.0 |
| RvD5_n-3 DPA_ | 361 | 199 | 0.5 | ± | 0.2 | 0.7 | ± | 0.3 | 0.8 | ± | 0.3 | 0.2 | ± | 0.1 | 0.4 | ± | 0.2 | 0.6 | ± | 0.2 |
|  |  |  |  |  |  |  |  |  |  |  |  |  |  |  |  |  |  |  |  |  |
| PD1_n-3DPA_ | 361 | 183 | 10.4 | ± | 2.6 | 10.6 | ± | 2.3 | 10.0 | ± | 2.3 | 9.5 | ± | 1.7 | 16.7 | ± | 7.4 | 9.8 | ± | 2.2 |
| 10S, 17S-diHDPA | 361 | 183 | 2.9 | ± | 1.3 | 2.3 | ± | 0.9 | 2.9 | ± | 0.9 | 13.1 | ± | 2.7 | 7.6 | ± | 2.5 | 10.9 | ± | 2.5 |
|  |  |  |  |  |  |  |  |  |  |  |  |  |  |  |  |  |  |  |  |  |
| MaR1_n-3 DPA_ | 361 | 249 | 0.5 | ± | 0.1 | 0.3 | ± | 0.1 | 0.4 | ± | 0.1 | 0.3 | ± | 0.1 | 0.9 | ± | 0.4 | 0.3 | ± | 0.1 |
| 7S, 14S-diHDPA | 361 | 249 | 1.7 | ± | 0.3 | 1.2 | ± | 0.2 | 1.6 | ± | 0.5 | 2.2 | ± | 0.6 | 2.6 | ± | 0.6 | 2.3 | ± | 0.5 |
|  |  |  |  |  |  |  |  |  |  |  |  |  |  |  |  |  |  |  |  |  |
| **EPA bioactive metabolome** |  |  |  |  |  |  |  |  |  |  |  |  |  |  |  |  |  |  |  |  |
| RvE1 | 349 | 195 | 2.1 | ± | 0.6 | 2.6 | ± | 0.8 | 2.4 | ± | 0.5 | 0.5 | ± | 0.1 | 1.1 | ± | 0.7 | 3.4 | ± | 2.6 |
| RvE2 | 333 | 199 | 11.3 | ± | 0.7 | 10.3 | ± | 0.9 | 12.3 | ± | 1.4 | 6.7 | ± | 0.7 | 12.1 | ± | 4.8 | 7.9 | ± | 0.9 |
| RvE3 | 333 | 201 | 0.2 | ± | 0.1 | 0.6 | ± | 0.2 | 0.5 | ± | 0.2 | 0.1 | ± | 0.0 | 0.8 | ± | 0.6 | 0.2 | ± | 0.1 |
|  |  |  |  |  |  |  |  |  |  |  |  |  |  |  |  |  |  |  |  |  |
| **AA bioactive metabolome** |  |  |  |  |  |  |  |  |  |  |  |  |  |  |  |  |  |  |  |  |
| LXA_4_ | 351 | 217 | 0.2 | ± | 0.0 | 0.2 | ± | 0.0 | 0.1 | ± | 0.0 | 0.1 | ± | 0.0 | 0.1 | ± | 0.0 | 0.1 | ± | 0.0 |
| LXB_4_ | 351 | 221 | 37.7 | ± | 8.1 | 42.0 | ± | 12.3 | 20.8 | ± | 4.8 | 7.9 | ± | 1.9 | 8.2 | ± | 1.2 | 7.2 | ± | 0.9 |
| 5S,15S-diHETE | 335 | 235 | 14.6 | ± | 1.9 | 15.7 | ± | 3.8 | 15.8 | ± | 3.0 | 10.5 | ± | 1.8 | 12.9 | ± | 4.5 | 7.8 | ± | 0.8 |
| 15R-LXA_4_ | 351 | 217 | 0.7 | ± | 0.1 | 0.8 | ± | 0.1 | 1.1 | ± | 0.3 | 0.3 | ± | 0.1 | 0.4 | ± | 0.2 | 0.3 | ± | 0.1 |
| 15R-LXB_4_ | 351 | 221 | 68.8 | ± | 11.8 | 76.1 | ± | 14.0 | 86.8 | ± | 14.1 | 39.9 | ± | 8.2 | 33.3 | ± | 6.7 | 34.0 | ± | 6.2 |
|  |  |  |  |  |  |  |  |  |  |  |  |  |  |  |  |  |  |  |  |  |
| LTB_4_ | 335 | 195 | 25.7 | ± | 5.1 | 20.1 | ± | 4.3 | 47.0 | ± | 17.6 | 4.9 | ± | 1.5 | 5.4 | ± | 2.1 | 23.1 | ± | 9.1 |
| 5S,12S-diHETE | 335 | 195 | 0.8 | ± | 0.5 | 0.8 | ± | 0.3 | 0.4 | ± | 0.2 | 0.3 | ± | 0.2 | 0.3 | ± | 0.1 | 1.4 | ± | 0.8 |
| 12-epi, Δ6-trans-LTB_4_ | 335 | 195 | 4.8 | ± | 0.9 | 4.6 | ± | 0.8 | 7.0 | ± | 2.6 | 0.6 | ± | 0.1 | 0.6 | ± | 0.2 | 3.3 | ± | 1.1 * |
| Δ6-trans-LTB_4_ | 335 | 195 | 4.8 | ± | 0.9 | 4.6 | ± | 0.8 | 6.9 | ± | 2.6 | 0.5 | ± | 0.1 | 0.8 | ± | 0.2 | 2.7 | ± | 0.8 * |
| 20-OH-LTB_4_ | 351 | 195 | 13.5 | ± | 3.2 | 10.2 | ± | 2.2 | 27.8 | ± | 8.8 | 40.1 | ± | 8.2 | 34.5 | ± | 6.6 | 36.3 | ± | 6.1 |
|  |  |  |  |  |  |  |  |  |  |  |  |  |  |  |  |  |  |  |  |  |
| LTC_4_ | 626 | 189 | 1.2 | ± | 0.5 | 1.3 | ± | 0.4 | 1.7 | ± | 1.3 | 0.5 | ± | 0.2 | 0.3 | ± | 0.1 | 0.4 | ± | 0.1 |
| LTD_4_ | 497 | 189 | 3.7 | ± | 1.4 | 13.5 | ± | 6.6 | 12.3 | ± | 3.9 | 5.7 | ± | 3.1 | 2.2 | ± | 0.5 | 9.3 | ± | 3.3 |
| LTE_4_ | 440 | 189 | 917.0 | ± | 303.0 | 1374.2 | ± | 495.1 | 1996.4 | ± | 905.7 | 103.6 | ± | 33.8 | 116.5 | ± | 26.7 | 458.6 | ± | 248.0 |
|  |  |  |  |  |  |  |  |  |  |  |  |  |  |  |  |  |  |  |  |  |
| PGD_2_ | 351 | 189 | 8.4 | ± | 1.3 | 10.1 | ± | 1.9 | 8.5 | ± | 1.2 | 7.1 | ± | 0.8 | 6.6 | ± | 1.7 | 5.7 | ± | 0.9 |
| PGE_2_ | 351 | 189 | 348.1 | ± | 82.0 | 227.6 | ± | 55.4 | 273.5 | ± | 54.1 | 36.2 | ± | 11.5 | 33.3 | ± | 11.8 | 23.7 | ± | 6.0 |
| PGF_2α_ | 353 | 193 | 705.2 | ± | 218.5 | 513.9 | ± | 154.4 | 455.0 | ± | 77.1 | 67.3 | ± | 17.4 | 71.3 | ± | 23.0 | 59.5 | ± | 15.8 |
| TxB_2_ | 369 | 169 | 121.9 | ± | 19.4 | 156.4 | ± | 21.5 | 198.5 | ± | 33.7 | 48.5 | ± | 8.0 | 27.9 | ± | 4.5 | 23.0 | ± | 12.0* |

† Results are in pg/ml of plasma. - = below limits; limits ~0.1 pg. Q1, M-H (parent ion); and Q3, diagnostic ion in the MS-MS (daughter ion). Results are expressed as pg/ml CSF and are mean ± s.e.m. n= 34 Day 0 placebo, 29 Day 0 81mg Aspirin, 29, Day 0 1000mg Aspirin, 34 Day 30 placebo, 27 Day 30 81mg Aspirin, 31 Day 30 1000mg Aspirin. * P, 0.05 vs Day 30 Placebo.
