# Supplementary material for: A randomised double blind placebo controlled phase 2 trial of adjunctive aspirin for tuberculous meningitis in HIV-uninfected adults
Source: eLife. 2018 Feb 27;7:e33478. doi: 10.7554/eLife.33478 (PMC5862527; doi:10.7554/eLife.33478)
Supplement: Supplementary file 7. [file elife-33478-supp7.docx]

**Study Title: A pilot phase II randomized controlled double blind trial of 81mg aspirin daily vs. 1000 mg aspirin daily vs. placebo as adjunctive therapy in HIV negative adults with tuberculous meningitis**

**Short Title: A pilot study of adjunctive aspirin for the treatment of HIV negative adults with tuberculous meningitis**

**Principal Investigators:**

Dr Nguyen Hoan Phu, Hospital for Tropical Diseases, Viet Nam

Dr Guy Thwaites, Oxford University Clinical Research Unit, Viet Nam

**Investigators:**

Dr Nguyen Thi Hoang Mai, Oxford University Clinical Research Unit, Viet Nam

Dr Doortje Heemskerk, Oxford University Clinical Research Unit, Viet Nam

Dr Tran Thi Van Thinh, Oxford University Clinical Research Unit, Viet Nam

Dr Nguyen Thuy Thuong Thuong, Oxford University Clinical Research Unit, Viet Nam

Dr Marcel Wolbers, Oxford University Clinical Research Unit, Viet Nam

Dr Ho Dang Trung Nghia, Pham Ngoc Thach Medical School, Viet Nam

Dr Tran Nguyen Tuyet Xuan, Hospital for Tropical Diseases, Viet Nam

Dr Tran My Phuong, Hospital for Tropical Diseases, Viet Nam

Dr Pham Phu Loc, Hospital for Tropical Diseases, Viet Nam

**TABLE OF CONTENTS**

[1 SYNOPSIS 4](#_Toc387306471)

[2 ABBREVIATIONS 6](#_Toc387306472)

[3 BACKGROUND AND RATIONALE 7](#_Toc387306473)

[4 OBJECTIVES 11](#_Toc387306474)

[4.1 Primary Objective 11](#_Toc387306475)

[4.2 Secondary Objectives 11](#_Toc387306476)

[5 TRIAL DESIGN 11](#_Toc387306477)

[5.1 Summary of Trial Design 12](#_Toc387306478)

[5.2 Primary and Secondary Endpoints/Outcome Measures 12](#_Toc387306479)

[6 Trial Participants 13](#_Toc387306480)

[6.1 Overall Description of Trial Participants 13](#_Toc387306481)

[6.2 Inclusion Criteria 13](#_Toc387306482)

[6.3 Exclusion Criteria 14](#_Toc387306483)

[6.4 Study Procedures 14](#_Toc387306484)

[6.4.1 Identification of Potential Participants 14](#_Toc387306485)

[6.4.2 Informed Consent 14](#_Toc387306486)

[6.4.3 Screening and Eligibility Assessment 15](#_Toc387306487)

[6.4.4 Baseline Assessments 16](#_Toc387306488)

[6.4.5 Randomisation and Dosing 16](#_Toc387306489)

[6.4.6 Subsequent Assessments 16](#_Toc387306490)

[6.5 Discontinuation of Treatment and Participation 17](#_Toc387306491)

[7 STUDY TREATMENT 18](#_Toc387306492)

[7.1 Description of Study Treatment 18](#_Toc387306493)

[7.2 Storage of Study Treatment 19](#_Toc387306494)

[7.3 Compliance with Study Treatment 19](#_Toc387306495)

[7.4 Accountability of the Study Treatment 19](#_Toc387306496)

[7.5 Prophylaxis 19](#_Toc387306497)

[7.6 Concomitant Medication 19](#_Toc387306498)

[7.7 Unblinding 20](#_Toc387306499)

[7.8 Post-trial Treatment 20](#_Toc387306500)

[7.9 Adverse events Management 21](#_Toc387306501)

[8 SAFETY REPORTING 21](#_Toc387306502)

[8.1 Definitions 21](#_Toc387306503)

[8.2 Assessment of Adverse Events 22](#_Toc387306504)

[8.3 Adverse Event Recording 24](#_Toc387306505)

[8.4 Regulatory Reporting of Adverse Events 24](#_Toc387306506)

[8.5 Safety Reporting and the Data Safety and Monitoring Board 24](#_Toc387306507)

[8.6 Protocol Violations 25](#_Toc387306508)

[9 STATISTICS 25](#_Toc387306509)

[9.1 Sample size justification 25](#_Toc387306510)

[9.2 Description of Statistical Methods 26](#_Toc387306511)

[10 DATA 27](#_Toc387306512)

[10.1 Data collection and entry 27](#_Toc387306513)

[10.2 Record Retention 28](#_Toc387306514)

[11 QUALITY CONTROL AND QUALITY ASSURANCE PROCEDURES 28](#_Toc387306515)

[12 ETHICS 28](#_Toc387306516)

[12.1 Ethical and Regulatory Guidelines 28](#_Toc387306517)

[12.2 Ethical Review 28](#_Toc387306518)

[12.3 Informed Consent 28](#_Toc387306519)

[12.4 Risks and Benefits 29](#_Toc387306520)

[12.4.1 Expenses and Benefits 30](#_Toc387306521)

[12.4.2 Participant Confidentiality 31](#_Toc387306522)

[12.5 Sample Use and Storage 31](#_Toc387306523)

[13 FINANCE AND INSURANCE 31](#_Toc387306524)

[14 PUBLICATION POLICY 31](#_Toc387306525)

[15 REFERENCES 31](#_Toc387306526)

[16 APPENDIX A: STUDY FLOW CHART 34](#_Toc387306527)

[17 APPENDIX B: SCHEDULE OF PROCEDURES 35](#_Toc387306528)

[18 APPENDIX C. TBM DIAGNOSTIC CRITERIA 36](#_Toc387306529)

[19 APPENDIX D. VIET NAM NATIONAL TUBERCULOSIS PROGRAMME TREATMENT GUIDELINES FOR TUBERCULOSIS MENINGITIS 37](#_Toc387306530)

# SYNOPSIS

| Study Title | **A pilot study of adjunctive aspirin for the treatment of HIV negative adults with tuberculous meningitis** |
| --- | --- |
| Internal ref. no. | 23TB |
| Clinical Phase | Phase II |
| Trial Design | Double blind, 1:1:1 randomized placebo controlled trial |
| Trial Participants | Adults with tuberculous meningitis |
| Planned Sample Size | 120 patients |
| Follow-up duration | 240 days |
| Planned Enrolment Period | September 2014 to December 2016 |
| Primary Objective | To generate pilot data on the safety and efficacy of 81mg daily and 1000mg daily dose adjunctive aspirin. |
| Secondary Objectives | To determine whether host genotype influences any of the above effects  To determine whether innate host macrophage antimicrobial activity influences outcome  To assess the plasma pharmacokinetics of aspirin |
| Primary Endpoint | Primary safety endpoint: Clinically significant upper gastro-intestinal or cerebral bleeding by 60 days  Primary efficacy endpoint: New MRI-proven brain infarction or death by 60 days |
| Secondary Endpoints | Time to death during 240 days  Neurological disability by 60 days and 240 days  Duration of hospital stay  Grade 3&4 and serious adverse events by 60 days  The proportion of patients with MRI-proven infarction by day 240  Antimicrobial activity of peripheral blood monocyte/macrophages on day 30 and 60 compared to baseline  Concentration-time profile of salicylic acid including exposure, peak levels, half-life, elimination clearance and volume distribution |
| Investigational Medicinal Products | Aspirin and placebo |
| Form | Tablet |
| Dose | 81mg,1000mg or placebo daily for 60 days |
| Route | Oral/nasogastric tube (if unable to swallow safely) |

#

# ABBREVIATIONS

| AE | Adverse event |
| --- | --- |
| ATC | Anti-tuberculosis chemotherapy |
| COX | Cyclooxygenase |
| CRF | Case Report Form |
| CSF | Cerebrospinal fluid |
| DSMB | Data Safety Monitoring Board |
| GCP | Good Clinical Practice |
| HIV | Human Immunodeficiency Virus |
| HTD | Hospital for Tropical Diseases, Ho Chi Minh City, Vietnam |
| ICF | Informed Consent Form |
| ICH | International Conference of Harmonisation |
| IMP | Investigational Medicinal Product |
| LTA4H | Leukotriene A4 hydrolase |
| MRI | Magnetic resonance imaging |
| NSAID | Non-steroidal anti-inflammatory drug |
| OUCRU | Oxford University Clinical Research Unit |
| OXTREC | Oxford Tropical Research Ethics Committee |
| PG | Prostaglandins |
| PI | Principal Investigator |
| PV | Protocol Violation |
| SAE | Serious Adverse Event |
| TBM | Tuberculous meningitis |
| TMF | Trial Master File |
| TXA2 | Thromboxane A2 |
| USAE | Unexpected Serious Adverse Event |

# BACKGROUND AND RATIONALE

Tuberculous meningitis (TBM) is the most devastating form of tuberculosis, killing or maiming around half of all sufferers [^1^](#_ENREF_1). There are three broad approaches to improving outcome: earlier diagnosis and treatment with anti-tuberculosis chemotherapy (ATC), enhancing anti-microbial killing by optimising or improving standard ATC, and controlling the intra-cerebral inflammatory response. Studies performed nearly a decade ago at the Hospital for Tropical Diseases (HTD), Ho Chi Minh City, demonstrated how simple clinical and laboratory methods assist in early TBM diagnosis [^2-4^](#_ENREF_2) and that adjunctive corticosteroids reduce mortality from the disease [^5^](#_ENREF_5). The current HTD/OUCRU trial (05TB) examines the hypothesis that enhanced early bacterial killing with high-dose rifampicin and levofloxacin will increase survival from TBM [^6^](#_ENREF_6). The trial completed recruitment of 817 adults with TBM in June 2014.

This pilot study returns to the long-standing hypothesis that outcome from TBM can be improved by control of the intra-cerebral inflammatory response. Our previous randomised controlled trial of adjunctive dexamethasone in 545 adults with TBM found dexamethasone increased survival but had no impact on neurological disability [^5^](#_ENREF_5). Indeed, 5-year follow-up of trial patients found dexamethasone’s impact on survival was lost after around 2 years, primarily because of the deaths of the higher proportion of severely disabled patients in the dexamethasone treatment arm[^7^](#_ENREF_7). There is, therefore, an urgent need to explore ways to prevent the neurological sequelae of TBM which complement the effect of dexamethasone on survival. We hypothesise that aspirin will reduce neurological sequalae from TBM by enhancing the resolution of intra-cerebral inflammation and by preventing intra-vascular thrombosis and brain infarction.

***Mechanism of TBM-related cerebral infarction***

Cerebral infarction is the commonest cause of irreversible neurological damage in patients with TBM, although the pathological mechanisms by which it occurs are poorly understood [^8^](#_ENREF_8). Post mortem studies performed in the late 19th century found that infarcts were most frequently seen in relation to small or medium-sized vessels occluded by intimal infiltration and/or proliferation [^9^](#_ENREF_9). Seminal autopsy and animal studies performed in the early 20^th^ century by Rich and McCordock reported vessel wall inflammation was more likely to have spread from the adventitia inwards, reflecting the severity of the surrounding subarachnoid inflammatory exudates [^10^](#_ENREF_10). In keeping with these observations, TBM-related infarcts are most commonly located in the territories of the proximal middle cerebral artery and the medial lenticulostriate and thalamoperforating vessels, where the basal meningeal inflammatory exudate is at its most intense [^8^](#_ENREF_8).

The role of vessel thrombosis in causing TBM-related cerebral infarcts is controversial. When specifically sought in autopsy TBM brains, some investigators have either failed to find arterial thrombosis, or found it to be uncommon [^11^](#_ENREF_11). Others assert that thrombosis is common, especially when there is an associated tuberculous vasculitis[^12^](#_ENREF_12). It seems most likely that TBM-related infarcts are caused by a variable combination of vasospasm, intimal proliferation and thrombosis [^8^](#_ENREF_8).

***Aspirin mechanism of action***

There are cogent reasons to believe that aspirin may reduce TBM-related infarcts and consequent neurological morbidity, when corticosteroids do not. Aspirin (acetylsalicylic acid) is the prototypical non-steroidal anti-inflammatory drug (NSAID), but one with unique anti-inflammatory and anti-thrombotic properties [^13^](#_ENREF_13). It acts by irreversibly inhibiting the cyclooxygenase (COX) pathway of arachidonic acid metabolism and the production of prostaglandins (PG). Aspirin is licenced in the United Kingdom at low dose (75-150mg) for the prevention of cerebrovascular disease (myocardial infarction and stroke, for example) and at higher dose (up to 4 grams daily) for the treatment of inflammatory conditions (e.g. arthritis and rheumatic fever).

Its anti-inflammatory effects are thought to occur at high dose (≥ 1 gram daily) and are produced by the inhibition of pro-inflammatory prostaglandins (e.g. PGE2, PGF2α and PGD2) as well as the unstable prostanoids, prostacyclin and thromboxane A2 (TXA2) [^13^](#_ENREF_13). Low dose aspirin (75-150mg) has less inhibitory effect on the pro-inflammatory prostaglandins, but still causes clinically significant inhibition of TXA2 and platelet aggregation. Until recently, it was the inhibitory effect on platelets and thrombus formation that was thought to explain aspirin’s well-documented reduction in the risk of death from stroke and myocardial infarction [^14^](#_ENREF_14). However, it is now appreciated that the beneficial effects of low dose aspirin may also stem from its ability to trigger the production of 15-epi-lipoxins, a class of molecules (alongside recently discovered resolvins, protectins and maresins) which actively promote resolution of inflammation[^15^](#_ENREF_15). The ‘pro-resolution’ properties of aspirin are not shared with any other NSAID and represents a potentially unique mode of action by which low-dose aspirin, alongside the prevention of thrombosis and stroke, might speed resolution of intra-cerebral inflammation and improve outcome from TBM.

***Clinical data supporting a role for aspirin in TBM treatment***

Two recent clinical studies suggest aspirin is safe when added to dexamethasone and may improve outcome from TBM. The first study randomised 118 Indian adults with TBM to standard anti-tuberculosis chemotherapy, with or without aspirin (150mg daily)[^16^](#_ENREF_16). The investigators hypothesised that aspirin would reduce the incidence of stroke and the primary endpoint was MRI-proven infarction by 3 months, with or without neurological deficit. By 3 months, MRI proven stroke occurred in 21 (33.3%) of all patients: 13 (43%) in the placebo arm and 8 (24%) in the aspirin group (P=0.18). Aspirin was associated with a significant reduction in mortality (43% versus 21.7%, P=0.02). The incidence of adverse events was not significantly different between the treatment groups and, in particular, there was no aspirin-associated gastro-intestinal bleeding reported. The results are hard to interpret, however, because of the variable use of prednisolone across the treatment arms. Prednisolone was given for severe disease at baseline, or if a patient’s condition worsened after the start of treatment. Any effect of aspirin, especially on mortality, is hard to disentangle from that of the corticosteroids.

A second study randomised 146 South African children with TBM to standard anti-tuberculosis chemotherapy plus placebo (n=50), low-dose aspirin (75mg/24 hours) (n=47), or high-dose aspirin (100mg/kg/24hours) (n=49) [^17^](#_ENREF_17). Aspirin has no significant impact on survival, but 9 children developed hemiplegia after the start of treatment and none of them received high-dose aspirin. Furthermore, the children in the high-dose aspirin group were significantly younger with more severe disease than the other treatment groups, yet their neurological outcomes were similar. Two children stopped aspirin early: one because of haematemesis and the other because of cerebral haemorrhage. Whether these patients received high or low dose aspirin was not reported.

***Leukotriene A4 hydrolase genetic polymorphisms and aspirin***

There is recent evidence from Vietnamese adults with TBM, that the balance of pro- and anti-inflammatory eicosanoids play an important role in determining susceptibility to TBM and the choice and impact of adjunctive inflammatory therapies. Using the zebra fish model of mycobacterial infection, Tobin and others found a polymorphism in the gene encoding the leukotriene A4 hydrolase (LTA4H) enzyme influenced tuberculosis susceptibility in both zebra fish and Vietnamese adults[^18^](#_ENREF_18). LTA4H controls the expression of pro- and anti-inflammatory eicosanoids, which in turn control the expression of the cytokine TNF-α (see **figure 1**).

**Figure 1. LTA4H genotype modulates susceptibility to mycobacterial infection**

*
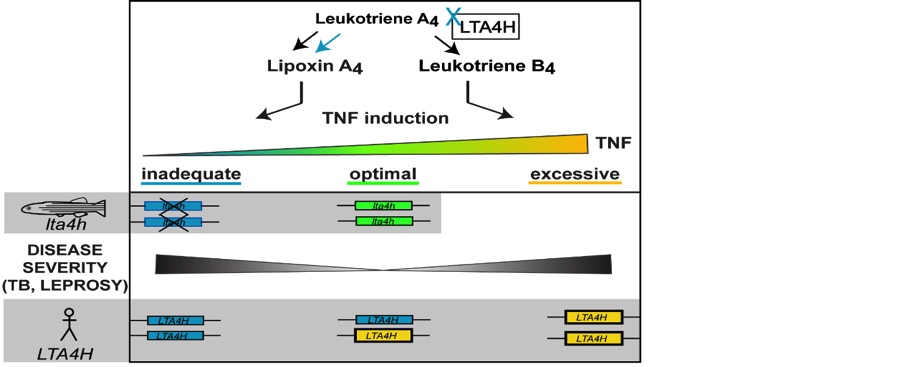
*

Zebra fish at both poles of the inflammatory response (minor and major allele homozygotes) were unable to control mycobacterial replication within granulomas, whereas those fish with an intermediate inflammatory response (the heterozygotes) controlled the infection. The phenotype of the homozygous fish could be changed to that of the heterozygotes through the respective addition of pro-inflammatory leukotriene B4 (LTB4) or TNF-α to LTA4H low fish, or by inhibition of TNF-α in the LTA4H high fish by either aspirin or dexamethasone [^19^](#_ENREF_19). Furthermore, in Vietnamese adults recruited to the earlier dexamethasone trial, only major allele homozygous adults with the hyper-inflammatory phenotype benefited from dexamethasone (**Figure 2**). Indeed, dexamethasone treatment of those with a hypo-inflammatory phenotype appeared to be detrimental.

**Figure 2. LTA4H genotype influences survival from TBM in all patients (A), pre-treatment CSF leucocyte counts (B), and survival in those who did not (C) and did (D) receive adjunctive dexamethasone.**


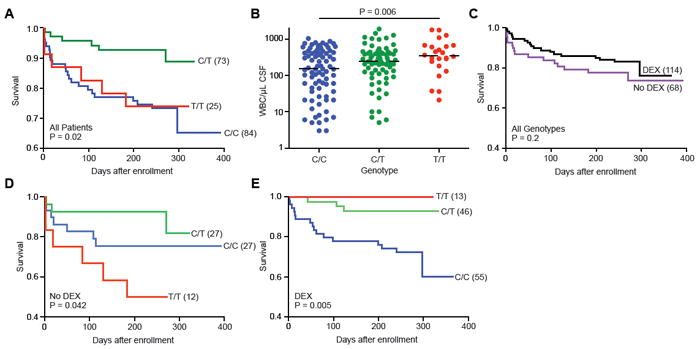


These findings need replication in a different population, and such studies are currently underway at OUCRU, but they provide an elegant potential explanation for the previous failure to find the mechanism by which corticosteroids improve survival from TBM in Vietnamese adults. They also have important implications for the current pilot study and we will investigate the influence of LTA4H genotype on aspirin’s effect on CSF inflammation and clinical outcome.

**Macrophage antimicrobial activity and outcome from TBM**

We and others have shown that polymorphisms in genes responsible for the innate immune response to *Mycobacterium tuberculosis* influence both susceptibility to TBM and disease severity ^20, 21^. Macrophages are the key effector cells in the innate immune response to *M. tuberculosis*, but how variations in their innate antimicrobial activity influence outcome from TBM have not been previously investigated. In this study we will apply a panel of functional assays to directly measure the rates of acquisition of a variety of lysosomal characteristics known to impact microbial survival ^22, 23^. We will investigate whether these characteristics change through treatment and how they relate to the patient’s outcome.

**Why do we need this pilot study?**

There are several outstanding questions that need to be addressed by a pilot study before embarking on a large, definitive, randomised controlled trial of adjunctive aspirin for TBM. These are:

1. What is the safety and tolerability of 81mg and 1000mg dose aspirin when added to dexamethasone for the first 60 days of treatment of TBM?
2. Does aspirin at these doses reduce MRI-proven infarction and neurological sequelae or death?
3. Does 81 mg aspirin daily have the same anti-inflammatory effect as 1000 mg aspirin daily (assessed by measurement of CSF prostaglandins) and which dose is likely to be most effective?
4. How does host genotype (in particular, LTA4H genotype) influence the anti-inflammatory, anti-thrombotic, and clinical effects of aspirin?

# OBJECTIVES

## Primary Objective

To generate pilot data on the safety and efficacy of 81mg daily and 1000mg daily dose adjunctive aspirin compared to no aspirin.

## Secondary Objectives

To determine whether host genotype, including LTA4H genotype, influences any of the above therapeutic effects of aspirin

To determine whether innate host peripheral blood monocyte/macrophage antimicrobial activity influences outcome from TBM and varies through treatment

To assess the plasma pharmacokinetics of aspirin

# TRIAL DESIGN

.

## Summary of Trial Design

The study is a parallel group, double blind, randomised, placebo controlled trial of 60 days of placebo vs. 81mg daily dose vs. 1000mg daily dose aspirin for the treatment of HIV-uninfected adults with tuberculous meningitis (see trial schema in appendix A). Patients will be recruited at the Hospital for Tropical Diseases (HTD), Ho Chi Minh City, Vietnam and should not have had more than 2 days of anti-tuberculosis chemotherapy or have received aspirin or any other NSAID within 2 weeks of randomisation. They should be able to have an MRI of their brain within 5 days of randomization.

Once enrolled, patients will be followed for 60 days and followed up at 8months for final outcome. They will all receive standard anti-tuberculosis chemotherapy and adjunctive dexamethasone (for 6-8 weeks), according to respective National Tuberculosis Programme guidelines (see attached guidelines for Viet Nam with this protocol). Participants will be stratified by site and MRC disease severity grade, and randomised on entry to one of three arms (1:1:1 ratio): aspirin 81mg, aspirin 1000mg or placebo daily for 60 days. Patients will be in-patients for at least the first 14 days of study treatment enabling real-time active surveillance of any adverse events after which they will be discharged according to clinical care. Study drug and standard treatment may be continued while patients are followed as out-patients.

Lumbar puncture will be performed before the start of treatment and then repeated in all patients on day 30 and 60 as per normal clinical care. Blood draws for pharmacokinetic assessment will be done 5 times between days 1 and 14. Assessment of peripheral blood monocyte/macrophage antimicrobial activity will be made at baseline and on day 240.

Clinical assessments will be performed at enrolment and weekly between day 7 and discharge. Brain magnetic resonance imaging (MRI) will be performed at baseline, day 60 and 240. A final clinical assessment of neurological disability will be made at 240 days.

## Primary and Secondary Endpoints/Outcome Measures

The primary safety endpoint will be the occurrence of clinically significant upper-gastro-intestinal bleeding and any cerebral bleeding confirmed by brain imaging by 60 days from randomisation.

Clinically significant upper gastro-intestinal bleeding will be defined as any one of the following:

1. Vomiting fresh or changed blood of any volume
2. Melena
3. Unexplained drop in haemoglobin concentration of >2g/L
4. Greater than 5mls of fresh or changed blood aspirated from nasogastric tube

The primary efficacy endpoint will be any new MRI-proven brain infarction or death by 60 days. To assess this endpoint MRI will need to be taken at baseline and at 60 days from randomisation. The scans will be read by an independent neuroradiologist blind to the treatment allocation.

The secondary endpoints will be:

- Number of grade 3&4 and serious adverse events by day 60 from randomisation
- Time to death during 240 days from randomisation
- Duration of hospital stay
- Neurological disability (as assessed by the modified Rankin score and Glasgow outcome score) by days 60 and 240.
- Resolution of CSF inflammation by day 30 through measurement of CSF leucocytes, protein, glucose, cytokines (TNF-α, IL-1β, IL-8, IL-10, IFNγ) and eicosanoids (15-epi-Lipoxin, Lipoxin A4, LTB4, PGE2, TBXB2, PGD2).
- Antimicrobial activity of peripheral blood monocyte/macrophages on day 240 compared to baseline.
- The proportion of patients with MRI-proven infarction by day 240
- Concentration-time profile of salicylic acid including exposure, peak levels, half-life, elimination clearance and volume distribution

# Trial Participants

## Overall Description of Trial Participants

The trial will enrol adults (≥ 18 years old) admitted to the Hospital for Tropical Diseases, Ho Chi Minh City. Patients should have a suspected diagnosis of TBM, as judged by the attending physician according to local guidelines, and require anti-tuberculosis treatment. Published diagnostic criteria will be used to categorise participants retrospectively into definite, probable, or possible TBM once the results of all investigations have returned (see appendix C)^24^.

## Inclusion Criteria

- Male or Female, aged 18 years or above.
- Suspected TBM and anti-tuberculosis chemotherapy either planned or started
- Less than 3 days of anti-tuberculosis chemotherapy taken for the current infection
- Patient or representative (if the patient is unable) is willing and able to give informed consent for participation in the study.

## Exclusion Criteria

Patients may not enter the study if ANY of the following apply:

- HIV infection (negative rapid test or Elisa test is required)
- They are unlikely, for any reason, to be able to have an MRI brain scan within 5 days (120 hours) of randomisation
- Known or suspected infection with multi-drug resistant tuberculosis (resistant to at least isoniazid and rifampicin)
- Unable to take isoniazid, rifampicin, or pyrazinamide at recommended doses for any reason
- History of diagnosed peptic ulceration or gastro-intestinal bleeding
- Active gastro-intestinal bleeding is suspected
- Taken >1 dose of aspirin (at any dose) or any other NSAID for any reason within 2 weeks of screening
- Aspirin considered mandatory for any reason by the attending physician
- Aspirin considered to be contraindicated for any reason by the attending physician
- Pregnancy or breast feeding (negative urine pregnancy test for all females of child-bearing age)
- Dexamethasone considered to be contraindicated for any reason by the attending physician .
- Any other significant disease or disorder which, in the opinion of the Investigator, may either put the participants at risk because of participation in the study, or may influence the result of the study, or the participant’s ability to participate in the study.

## Study Procedures

### Identification of Potential Participants

All patients with suspected TBM according to local guidelines will be identified for screening by the ward doctors. As a part of clinical care patients will have a lumbar puncture, and when possible, a GeneXpert MTB/RIF test on CSF to assess likelihood of infection with multi-drug resistant tuberculosis. The results of these tests will be available for study screening.

### Informed Consent

Informed consent will be taken by the attending doctors, all of whom will receive specific training in the study and will be authorised to take consent by the trial PI. These doctors will also assess whether or not the patient has mental capacity to provide informed consent. If the doctor judges that the patient does not have this capacity (for any reason, but most likely to be confusion or coma secondary to TBM), they will obtain informed consent from the patient’s representative (usually a relative).

It must be made completely and unambiguously clear that the patient (or their representative) is free to refuse to participate in all or any aspect of the trial, at any time and for any reason, without incurring any penalty or affecting their treatment.

The informed consent form will be presented to the participants or representatives detailing no less than: the exact nature of the study; the implications and constraints of the protocol; the known side effects, risks involved and alternatives to taking part. Those who refuse consent will be treated as per the best available standard of care and will not have any study related procedures performed.

The patient or their representative must personally sign and date two of the latest approved version of the informed consent form. The study staff will also sign and date the two copies.

If the patient/representative is illiterate, a witness who is not a member of the study staff will be present during the informed consent discussion. The informed consent form will be read to the patient/representative in the presence of the witness. If the patient/representative agrees to participate, the form will be signed and dated by the witness.

If consent is provided by a representative and the patient regains the capacity to consider participation during the study period, the patient should be consulted and informed consent to continue the study obtained. If the patient refuses to give informed consent to participate s/he will be withdrawn from the study without compromise to their clinical care.

### Screening and Eligibility Assessment

Potential participants will be screened by the attending physicians. Screening will include the CSF analysis results of the standard diagnostic lumbar puncture and GeneXpert MTB/RIF test when available. Any residual volume of CSF remaining from this lumbar puncture will be stored for the purposes of this study. The results of an HIV test (performed routinely on all patients admitted to the ward) will also be available at screening. Results of any tests performed for clinical care during this illness episode may be used for the purposes of screening. Residual volumes of samples drawn for clinical care during this illness episode may be tested or stored for analysis detailed in this protocol. Results of tests on cerebrospinal fluid obtained within 7 days of randomization and results on blood obtained within 2 days of randomization may be applied to meet inclusion/exclusion criteria.

A screening log will be kept on the ward, with a record of all patients screened and how they met/did not meet the study entry and exclusion criteria. Patients who do not meet the study criteria will be informed as such and treated as per best available clinical care.

### Baseline Assessments

Eligible patients will be carefully assessed clinically at baseline, with special attention paid to the duration of illness, Glasgow coma score, and focal neurological deficit. The results of all diagnostic lumbar punctures and relevant tests will be recorded. MRI brain should be performed as soon as possible and within 5 days of randomisation if not previously performed within the 5 days prior to randomisation.

Blood (5mls) will be taken from all patients and stored for later testing of host genetic polymorphisms which may influence disease pathogenesis and response to treatment, including LTA4H. Patients will have 20mls heparinised blood taken at baseline for isolation of peripheral blood mononuclear cells and the subsequent assessment of the antimicrobial activity of the monocytes/macrophages.

If the patient has not already started anti-tuberculosis drugs, these should be initiated immediately along with adjunctive dexamethasone (recommended for all patients with TBM). The clinical condition of the patient, together with the results of prior CSF analysis should be recorded in the clinical record form (CRF).

### Randomisation and Dosing

Randomisation will be 1:1:1 to 81mg aspirin, 1000mg aspirin and placebo, with stratification by MRC disease severity grade. Subject numbers will be stratified and assigned sequentially as each subject is randomised. Each patient will receive the next sequential package of blinded study drugs which will be prepared in advance and available on the ward. Each treatment pack will contain two bottles of study treatment. One bottle will contain 81mg tablets of aspirin or placebo, the second bottle will contain 500mg tablets of aspirin or placebo. For 60 days, patients will take one 81mg tablet and two 500mg tablets. Administration of the two daily 500mg tablets will be separated by approximately 12 hours.

The first dose of one 81mg tablet and one 500mg tablet will be given as soon as possible after randomisation. One 500mg tablet will be taken approximately 12 hours later. In the case of the first dose being taken late in the day on study day one, the second dose may be given over a shorter time period to ensure the full dose is administered on the first day.

Patients will take medication orally with water if required. Patients unable to swallow will be given crushed tablets via a naso-gastric tube at the same doses.

Patients who vomit within 30 minutes of dosing will be given a replacement dose.

### Subsequent Assessments

The outline of study assessments is provided in appendix B.

Follow-up clinical assessments should be made weekly until discharge from hospital. The assessments will include careful neurological examination and proactive surveillance for symptoms and signs of aspirin toxicity/adverse reactions (e.g. gastritis, tinnitus, gastrointestinal bleeding, renal impairment). All adverse events will be documented, followed and assessed for the likelihood of being related to aspirin.

A full blood count, and measurement of plasma urea, creatinine, electrolytes and stool microscopy for red blood cells will be taken on each clinical assessment day, day 30 and day 60 (5mls EDTA blood; 5mls clotted blood). Patients will have 20mls of heparinised blood taken at day 240 for assessment of the antimicrobial activity of peripheral blood monocytes/macrophages. Whole blood (2ml) will be obtained at 5 time points during the first 14 days of study treatment and analysed to assess the pharmacokinetic characteristics of aspirin.  Time points will be randomly assigned to a range of periods after drug dosing. A concentration-time profile of salicylic acid including exposure, peak levels, half-life, elimination clearance and volume distribution will be analysed.

Patients will have lumbar punctures on days 30 and 60, as per normal clinical care of patients with TBM in the hospital. 10mls of CSF should be taken when possible (as per normal care) and standard biochemical and microbiological tests performed (including mycobacterial smear and culture and GeneXpert MTB/RIF on all specimens collected during the study period). The CSF supernatant should be stored immediately at -80^0^C for later analysis of cytokine and eicosanoid concentrations.

A full clinical examination, brain MRI and neurological disability assessment by the Glasgow coma score and the Modified Rankin score will performed on days 60 and 240.

Window periods for all study procedures will be: +/- 2 days for any procedure scheduled for day 7, 14, 21, 28; +/- 5 days for any procedure scheduled on day 30 – 60; and +/- 14 days for any procedure scheduled for day 240. Screening, enrolment and day 1 schedule windows are as detailed in the appropriate sections of this document.

Published diagnostic criteria will be applied to all enrolled patients at discharge or death, and when all mycobacterial culture results are available (see appendix C). The criteria will sub-divide all cases into definite, probable and possible tuberculous meningitis, and those with an alternative diagnosis.

## Discontinuation of Treatment and Participation

If a patient or the representative who has given consent on their behalf, chooses to discontinue trial treatment, they should be followed up (providing they are willing) and encouraged to follow the study procedures in lieu of withdrawing from the trial. If they do not wish to remain on trial follow-up, however, their decision will be respected and the patient will be withdrawn from the trial completely. This will be recorded on the OUTCOME CRF. The reason for the patient withdrawing should be ascertained wherever possible. Prior to withdrawing completely from the trial, the patient will be invited to have assessments performed as appropriate for the final visit although they would be at liberty to refuse any or all individual components of the assessment.

In addition, the investigator may discontinue the study drug at any time the investigator considers it necessary for any reason including those below. These patients will continue to be followed according to the study protocol.

- A serious adverse event related to aspirin
- Pregnancy
- Ineligibility (either arising during the study or retrospective having been overlooked at screening e.g. an alternative diagnosis to TBM being confirmed)
- Significant protocol violation
- Significant non-compliance with treatment regimen or study requirements

# STUDY TREATMENT

## Description of Study Treatment

An independent trial pharmacist will generate a randomization list stratified by site and MRC grade, with treatment assignments in blocks of varying size. Based on this list, identical treatment packs will be created and distributed to the wards. Each treatment pack will contain two bottles. The first bottle will contain 60 tablets of either 81mg aspirin or matched placebo. The second bottle will contain 120 tablets of either 500mg aspirin or matched placebo. Additional pills will be added to bottles to allow for accidental discards, replacement of vomited doses and to limit manipulation of compliance pill counts. All patients will take one 81mg tablet and two 500mg tablets per day. Administration of the two daily 500mg tablets will be separated by approximately 12 hours. Treatment allocation will be as follows:

Arm A: 81mg aspirin – one tablet 81mg aspirin, two tablets 500mg-matched placebo (bottle #1 is aspirin, bottle #2 is placebo)

Arm B: 1000mg aspirin – one tablet 81mg-matched placebo, two tablets 500mg aspirin (bottle #1 is placebo, bottle #2 is aspirin)

Arm C: placebo – one tablet 81mg-matched placebo, two tablets 500mg-matched placebo (bottle #1 and #2 are placebo)

Daily treatment of one 81mg size tablet and two 500mg size tablets (1000mg in two divided doses) will be taken for 60 days.

The 81mg tablets and 500mg tablets will not be visually matched, hence the need for patients to take both tablets for each dose. All tablets will be coated to prevent taste differentiation between active drug and placebo.

## Storage of Study Treatment

Aspirin and placebo are stable at room temperature for 24 months.

## Compliance with Study Treatment

For the first 14 days of treatment, the drug will be administered under observation of the ward nurses thus ensuring compliance with the regimen. Patients may be discharged after day 14 and may complete study drug treatment at home. Compliance inquiry and counselling will be done at each study visit by the study staff. Patients will be instructed to return all medication and bottles at the day 60 visit when a pill count will be done. Medication bottles of patients who die or withdraw will be collected when possible for compliance and inventory checks.

## Accountability of the Study Treatment

All receipt, transfers, dispensing, administration and return of study drug will be accounted for by the trial pharmacist. Study drug will be prescribed and administered by standard hospital and OUCRU procedures. Ward nurses trained in study procedures will be responsible for safe storage and in-hospital administration.

## Prophylaxis

Prophylaxis against upper gastro-intestinal symptoms and bleeding is recommended. Patients will be prescribed ranitidine (300mg at night) unless the attending physician decides otherwise. Antacids should be avoided, if possible, as they may increase the urinary excretion of aspirin. Proton pump inhibitors (e.g. omeprazole) should also be avoided as they interact with rifampicin.

## Concomitant Medication

Throughout the study, Investigators may prescribe any concomitant medications or treatments deemed necessary to provide adequate supportive care, with the exception of other NSAIDS.

Study doctors should also be aware of the following potential interactions noted in the aspirin summary of produce characteristics (SPC):

- Aspirin may enhance the effects of anticoagulants and oral hypoglycaemic agents.
- Other antiplatelet drugs such as clopidogrel and ticlopidine increase the risk of bleeding.
- Aspirin may enhance the effects of phenytoin and sodium valproate.
- The activity of methotrexate may be markedly enhanced by aspirin and its toxicity increased
- Aspirin may antagonise the diuretic effect of spironolactone and may reduce acetazolamide excretion (risk of toxicity).
- Aspirin increases plasma concentration of zafirlukast.
- Metoclopramide and domperidone enhance the effect of aspirin (increased rate of absorption).
- Avoid concomitant administration with mifepristone (theoretical interaction).
- Aspirin may inhibit action of uricosurics.
- The toxicity of sulphonamides may also be increased by aspirin.
- Aspirin is pharmaceutically incompatible with iron salts and alkalis.

Any toxicity relating to anti-tuberculosis drugs will be managed according to national guidelines (see national guidelines in Appendix D) or treatment procedures agreed by the hospital.

## Unblinding

Unblinding means revealing the identity of the study treatment (i.e. aspirin or placebo). Study treatment should only be unblinded if knowing the treatment that a patient has been allocated will result in a change in the patient’s management.

The decision whether or not to unblind should be discussed with the Principal Investigator when possible. Unblinded treatment allocation information will be available in opaque, tamper-proof envelopes held securely at each site and available at all times. The responsibility to approve unblinding will be assigned to dedicated site staff. Access to treatment allocation information should only be given with the approval of one of these dedicated staff. Unblinding will be documented in the case record form.

## Post-trial Treatment

Study medication will not be continued beyond the trial period of 60 days. Participants will continue standard care throughout the trial period and after the study treatment period is complete. Anti-tuberculosis treatment will be given thereafter for at least 6 months in line with national tuberculosis treatment guidelines. There are no restrictions on concomitant medicines once study treatment has stopped.

## Adverse events Management

Study treatment will be discontinued for all grade 3 and 4 events that are considered related to Aspirin use. For an event to be considered related it must follow a reasonable temporal sequence from trial medication administration and not reasonably be attributed to any other cause. Recognised adverse reactions to aspirin are listed in section 8.2 below. Study drugs will not be re-introduced in patients who have stopped treatment due to an adverse event. Management of adverse events will follow normal care and current practice guidelines.

# SAFETY REPORTING

## Definitions

**Adverse Event (AE)** - is any untoward medical event that occurs to a study participant during the course of the study whether or not that event is considered related to the study drug. An AE can, therefore, be any unfavorable and unintended sign (including an abnormal laboratory finding, for example), symptom, or disease temporally associated with the study drug, whether or not considered related to the study drug.

Stable chronic conditions, such as arthritis, which are present prior to clinical trial entry and do not worsen are not considered AEs and will be documented in the subject’s clinical chart as medical history.

Clinical or laboratory events are considered adverse events only if they occur after the first dose of study treatment (see below for repor ting of adverse events).

Serious Adverse Events - An AE is considered to be "serious" if it results in one of the following outcomes

- Death,
- Life-threatening event (the subject was at immediate risk of death at the time of the event; it does not refer to an event which hypothetically might have caused death if it were more severe),
- Inpatient hospitalization (new admissions) or prolongation of existing hospitalization (beyond what is expected for normal clinical care)
- Persistent or significant disability/incapacity (a substantial disruption of a person's ability to conduct normal life functions),
- Congenital anomaly/birth defect

Any pregnancy which occurs during the trial treatment period will be followed until outcome. Any congenital abnormality or birth defect will be recorded as a serious adverse event.

**Unexpected Serious Adverse Events** - are untoward medical events which fit one or more of the criteria for SAEs above and which are not considered a part of normal clinical progression of disease or an expected reaction to standard treatment therapy. Any event that becomes of concern to the investigators or study doctors during the course of the trial may be reported as a USAE.

## Assessment of Adverse Events

Adverse events will be graded according to the Common Terminology Criteria for Adverse Events (CTCAE) definitions: <http://ctep.cancer.gov/protocolDevelopment/electronic_applications/ctc.htm>. In the event that an adverse event is not described within the CTCAE definitions, the following generic severity grading will be used:

**Grade 1 Mild;** asymptomatic or mild symptoms; clinical or diagnostic observations only; intervention not indicated.

**Grade 2 Moderate;** minimal, local or noninvasive intervention indicated; limiting age-appropriate instrumental Activities of Daily Living (ADL)*.

**Grade 3 Severe or medically significant but not immediately life-threatening;** hospitalization or prolongation of hospitalization indicated; disabling; limiting self care ADL**.

**Grade 4 Life-threatening consequences**; urgent intervention indicated.

*Instrumental ADL refer to preparing meals, shopping for groceries or clothes, using the telephone, managing money, etc.

**Self-care ADL refer to bathing, dressing and undressing, feeding self, using the toilet, taking medications, and not bedridden.

[Note: “Life-threatening” as a severity grade is not necessarily the same as “life-threatening” as a “serious” criterion used to define a serious adverse event. The former is a “potential” threat to life and the latter is an “immediate” threat to life.]

A laboratory abnormality only needs to be recorded as a clinical adverse event if it is associated with an intervention. Intervention includes, but is not limited to, discontinuation of a current treatment, dose reduction/delay of a current treatment, or initiation of a specific treatment. In addition, any medically important laboratory abnormality may be reported as an adverse event at the discretion of the investigator. This would include a laboratory result for which there is no intervention but the abnormal value suggests disease or organ toxicity. Laboratory events will be graded according to CTCAE definitions.

If clinical sequelae are associated with a laboratory abnormality, the diagnosis or medical condition should be reported as the adverse event (e.g., renal failure, haematuria) - not the laboratory abnormality (e.g., elevated creatinine, urine RBC increase).

The relationship of each adverse event to the trial medication must be determined by a medically qualified individual according to the following definitions:

**Related**: The adverse event follows a reasonable temporal sequence from trial medication administration. It cannot reasonably be attributed to any other cause.

**Not Related**: The adverse event is probably produced by the participant’s clinical state or by other modes of therapy administered to the participant.

Patients will be under active surveillance for adverse events. All adverse events will be scored as not related, possibly related, or related to the study drug. The events listed below, and any others agreed between the treating doctor and Principal Investigator, are recognised adverse reactions to aspirin may be considered related to the trial intervention:

**Blood and lymphatic system disorders** - anaemia, haemolytic anaemia, hypoprothrombinaemia, thrombocytopenia, aplastic anaemia, pancytopenia, prolonged bleeding time, occult blood loss, elevated transaminase levels, agranulocytosis.

**Gastrointestinal disorders** - gastrointestinal bleeding, erosions, perforations or ulceration which can occasionally be major (may develop bloody or black tarry stools, severe stomach pain and vomiting blood), gastrointestinal irritation (mild stomach pain, heartburn, vomiting and nausea).

**Hepatic disorders** - hepatitis (particularly in patients with SLE or connective tissue disease)

**Renal and urinary disorders** – disturbances of renal function

**Ear and labyrinth disorders** - tinnitus

**Salicylism**- mild chronic salicylate intoxication may occur after repeated administration of large doses, symptoms include dizziness, tinnitus, deafness, sweating, nausea, vomiting, headache and mental confusion.

**General disorders and administration site conditions** – Allergic reactions: rhinitis, urticaria, purpura, Stevens-Johnson syndrome, angioneurotic oedema, angio-oedema, asthma, worsening of asthma, bronchospasms.

## Adverse Event Recording

All adverse events which initiate after administration of the first dose of study drug and before the end of study day 60 will be recorded in the CRF. Events which are grade 3 and 4 adverse events and those judged to be possibly related or related to the trial intervention will be followed to resolution.

## Regulatory Reporting of Adverse Events

As serious adverse events and mortality are common in tuberculous meningitis, safety reporting will focus on events of potential relevance to the trial intervention. The following events will be reported to the site ethical committee and the ethical committee of the Viet Nam Ministry of Health:

- All unexpected serious adverse events
- All serious adverse events judged to be related or possibly related to the trial intervention
- All deaths

Reporting to the ethical committees will include an initial written report, which will be sent as soon as possible and within 7 days of occurrence. The format and content of the initial report should follow the Viet Nam Ministry of Health report template and include all information available at the time of reporting. A follow up report with complete details will be sent within 15 days of the initial report if the initial report does not contain the details of event resolution.

## Safety Reporting and the Data Safety and Monitoring Board

An independent Data and Safety Monitoring Board will be established consisting of expert Vietnamese and international researchers and doctors, with the necessary clinical, research and statistical knowledge. The DSMB will review the protocol and agree to a data review schedule and reporting requirements applicable before the study commences, with particular reference to the details of the interim review. A DSMB charter will outline its responsibilities and how it will operate.

The DSMB will perform a safety review of data for the first 30 patients enrolled to the trial. This review will include unblinded summary tables of baseline characteristics, SAEs, AEs and event reports submitted to the DSMB. An analysis of overall clinical outcome will be performed. If deemed necessary, an additional safety review may be performed after the enrolment of 60 patients or annually at the discretion of the DSMB based on available data and ongoing reporting. All DSMB reports will be sent to the responsible ethical committees including the site ethical committees, the Oxford Tropical Research Ethics Committee and the Viet Nam Ministry of Health ethical committee for consideration. Recruitment will continue at any active site during the DSMB review period.

As the dissemination of preliminary summary data could influence the subsequent conduct of the trial and introduce bias, access to interim data and results will be confidential and strictly limited to the DSMB members. No results (except for the recommendation) will be communicated to the outside and/or the clinical investigators involved in the trial.

## Protocol Violations

Protocol violations (PV) are events that contradict or omit protocol instructions and fulfil one or more of the following criteria: 1) the safety/welfare of one or more patients is put at risk by the non-compliance, 2) the integrity of study data is compromised by the violation.

PVs must be reported to the sponsor as soon as possible. If the PI or sponsor confirms that the PV poses a risk to patient safety/welfare or the integrity of study data, the PV must be reported to the responsible hospital Ethical Committee.

# STATISTICS

## Sample size justification

Sample size was chosen based on clinical and feasibility considerations. The calculations below provide evidence of what can be achieved with a sample size of 40 patients per group for the primary safety and efficacy endpoints, respectively.

The proportion of HIV uninfected patients with clinically significant gastro-intestinal bleeding from our on-going 05TB trial of hyper-intensive anti-tuberculosis chemotherapy in Vietnam is about 1% (3/278). Cerebral bleeding associated with TBM is extremely rare (estimated at <0.01% of all patients), but it is possible it may be more common in those treated with aspirin. We have, therefore, included in the primary assessment of safety.

Assuming no effect of aspirin on gastrointestinal bleeding, i.e. a true gastrointestinal bleeding risk of 1% for aspirin, the probability will be 67% to observe 0/40 patients and 27% to observe 1/40 patient experiencing gastrointestinal bleeding in each aspirin arm. The corresponding upper limits of the 95% confidence intervals for the risk of a gastrointestinal bleed would allow “proving” that the true gastrointestinal bleeding risk for aspirin is (at worst) ≤8.8% (in case of 0/40 observed gastrointestinal bleeding) or ≤12.9% (in case of 1/40 observed gastrointestinal bleeding).

Assuming further equal gastrointestinal bleeding risks of 1% in the combined aspirin groups, the probability will be 80% that we observe zero or one bleeding event amongst the 80 patients receiving aspirin and the corresponding upper limit of the 95% confidence interval for the risk of a bleed would allow “proving” that the true overall bleeding risk for aspirin is (at worst) ≤6.8%.

If in contrast, the risk of bleeding was increased to 10% in one of the aspirin arms, the probability would be 78% that we observe 3/40 patients with gastrointestinal bleeding or more with a corresponding 95% confidence interval that would “prove” that the true bleeding risk for that aspirin arm were (at best) ≥2.5%.

We expect a risk of new MRI-proven brain infarction or death within 60 days of approximately 40% in the control arm. Based on the results of the trial performed by Misra et al ^16^, we assume that 81 mg aspirin daily may halve this risk to 20% and the risk in the 1000 mg aspirin daily arm is between 20-40%, then a linear trend test across the 3 groups will have approximately 75% power to detect such an effect at the one-sided 10% significance level (i.e. to generate “mild evidence”) and approximately 50% power to detect it at the conventional one-sided 2.5% significance level.

## Description of Statistical Methods

The risk of clinically significant upper-gastro-intestinal bleeding and image-proven cerebral bleeding by 60 days will be summarized as x/n (%) in each group together with two-sided 95% Wilson confidence intervals and corresponding confidence intervals for risk differences between groups.

The risk of the primary efficacy endpoint of new MRI-proven brain infarction or death by 60 days will be summarized in the same way. In addition, we will perform a linear trend test for group differences based on logistic regression with adjustment for gender, MRC grade, and age.

The risk of disability or death will be summarized as for the primary efficacy endpoint and the analysis of overall survival during 240 days will be based on Kaplan-Meier graphs and Cox regression. The frequency of adverse events in each study arm and resolution of CSF inflammation will be analysed descriptively.

Analyses to explore the effect of LTA4H genotype will include this information as an additional covariate in the regression models.

As this is a pilot study of relatively small size and limited power, p-values will be interpreted with caution and a one-sided p-value <0.1 will be regarded as mild evidence for an effect. Corresponding two-sided 95% confidence intervals will be provided for all tests to assess the range of plausible magnitudes of effects given the data.

No imputation of missing data will be performed. For the analysis of GI/cerebral bleeding by day 60, patients lost to follow-up before day 60 (without prior GI bleeding) will be excluded from the analysis to get a conservative estimate of the true proportion of bleeding in the aspirin groups. The analysis of the primary efficacy endpoint will exclude patients with missing baseline or follow-up MRI scans (except for prior death).

All analyses will analyze patients according to their randomized treatment group.

# DATA

## Data collection and entry

Source documents will be generated during the study by the site ward and study staff. Source documents include all original recordings of observations or notations of clinical activities, and all reports and records necessary for the evaluation and reconstruction of the clinical trial. Source documents include, but are not limited to, the subject’s medical records, research case record forms (paper or electronic), laboratory reports, MRI data, radiologist’s reports, progress notes, pharmacy records, and any other similar reports or records of procedures performed during the subject’s participation in the study.

Access to applicable source documents is required for study purposes. The site investigators are responsible for maintaining any source documentation related to the study. Source documentation should support the data collected on the CRF when the CRF is not the original site of recording, or else the reason for the difference documented. Source documentation must be available for review or audit by the sponsor or designee and any applicable regulatory authorities.

Case Report Forms (CRFs) will be used as a data collection tool. CRFs may be used as source documents if they are the primary data collection tool for specified data as documented in written standard operating procedures. The site Investigators are responsible for maintaining accurate, complete and up-to-date records. These forms are to be completed on an ongoing basis during the course of the study by authorized individuals.

Corrections to paper CRFs must be initialled and dated by the person making the correction and must not obliterate the original entry. All CRFs should be reviewed by the designated study staff and signed as required with written or electronic signature, as appropriate.

Selected study members will be trained on how to enter all clinical data as source information from the CRFs and from laboratory source documents into an internet-based computerized data entry system called CliRes hosted by OUCRU. Source documents and electronic data will be verified according to the Data Management Plan and Trial Monitoring Plan.

## Record Retention

The investigator is responsible for retaining all essential for at least 15 years after the completion of the trial. Original paper documents will be maintained for a minimum of 5 years and electronic documents retained thereafter. All stored records are to be kept secure and confidential.

# QUALITY CONTROL AND QUALITY ASSURANCE PROCEDURES

The study will be conducted in accordance with the current approved protocol, ICH GCP, relevant regulations and standard operating procedures.

Regular monitoring will be performed according to ICH GCP by OUCRU CTU. Data, drugs, samples and procedures will be evaluated for compliance with the protocol, standard operating procedures, regulatory requirements and terms of ethical approval. Records will be verified for accuracy against source documents and physical inventory of drugs and samples.

# ETHICS

## Ethical and Regulatory Guidelines

The Investigator will ensure that this study is conducted in accordance with the principles of the Declaration of Helsinki (Seoul 2008) and the terms of approval of the appropriate ethical committees.

The Investigator will ensure that this study is conducted in full conformity with relevant regulations and with the ICH Guidelines for Good Clinical Practice July 1996.

## Ethical Review

This protocol and all associated informed consent forms will be submitted to the ethical committee of the Hospital for Tropical Diseases and the Viet Nam Ministry of Health. The Investigators will submit and, where necessary, obtain approval from the above parties for all substantial amendments to the original approved documents.

## Informed Consent

Written informed consent will be obtained from all study participants or their representatives before any study interventions take place.

The study staff will discuss the study with all potential adult patients or, in the case of a patient who is unable to give informed consent independently, with an appropriate representative. Study staff will describe the purpose of the study, the study procedures, possible risks/benefits, the rights and responsibilities of patient, and alternatives to enrolment. The patient or representative will be invited to ask questions which will be answered by study staff, and they will be provided with appropriate numbers to contact if they have any questions subsequently. If the patient or representative agrees to participate, they will be asked to sign and date two copies of an informed consent form.  A copy of the form will be given to them to keep.  If required, the patient or representative will be given up to 24 hours to consider the study provided the patient remains eligible for the study.

Patients who were consented by a representative will be approached to consider consent independently if at any time during study participation s/he becomes able to consider consent independently.

In addition to the procedures above, illiterate signatories will have the informed consent form read to them in the presence of a witness who will sign to confirm that the form was read accurately and that the participant or representative agrees to participation. All informed consent forms will be written in the local language and will use terms that are easily understandable. Clinical care will not be delayed in any case during consideration of consent.

## Risks and Benefits

All patients will receive the best available standard-of-care anti-tuberculosis chemotherapy in addition to adjunctive dexamethasone. The anti-tuberculosis drugs have no adverse interactions with aspirin. The addition of aspirin to dexamethasone therapy may increase the risk of gastro-intestinal bleeding, hence the pilot nature of this study. Furthermore, it is possible aspirin may increase the risk of cerebral bleeding, although the overall risk of TBM-related cerebral bleeding is considered extremely low (estimated <0.01%). Previous trials of aspirin for TBM, although involving relatively small numbers of patients (n=264), have not reported an increased incidence of bleeding of any time. Nevertheless, we need to know whether aspirin at dose of 81mg or 1000mg is safe in this context before larger trials can be contemplated.

Otherwise, there are few additional risks to patients and nearly all follow-up investigations (including blood tests and repeated lumbar punctures) are part of the normal standard of care for patients with TBM in HTD. Only sequential brain MRIs are not routinely performed, but these are very low risk as they do not expose the patients to radiation.

Against these risks, trial patients may benefit from receiving aspirin by its ability to control intra-cerebral inflammation and reduce the risk of TBM-associated stroke. In addition, all patients will benefit from the careful observation and follow-up from enrolment, which will allow the complications of TBM to be rapidly identified and managed.

The risks and benefits of participation will be communicated in two ways. First, all potential patients or their representatives will be given an infomred consent form clearly listing the risks and benefits of the trial. Second, all potential patients (or their representatives) will be able to discuss participation with their physician who will be able to address questions not covered or arising from the patient information sheet.

The trial protocol will seek ethical approval to include incapacitated/comatose adults in the trial as we consider many of these adults will have the most severe infection and therefore represents the group that might stand most to gain from aspirin. We anticipate around 50% of patients be critically ill with reduced consciousnnes and unable to consent for themselves. Once incapacity has been confirmed by one of the ward physicians, written informed consent will be sought from the patient’s relative/representative. If the subject regains capacity during treatment they will be informed of the consent given by their representative and their wishes respected concerning on-going participation. If they are happy to remain in the trial, the patient should sign and date two copies of an informed consent form at this time. If they wish to withdraw from the trial, no further trial-related procedures will be performed, but data to this point would be used in analysis. Data from any patient who dies before regaining capacity (but whose representative has provided consent) will be included in analysis.

Patients’ confidentiality will be maintained throughout the trial. Data submitted to OUCRU Clinical Trials Unit and samples sent to central testing facilities will be identified only by the trial number and patient initials.

### Expenses and Benefits

The study funding will cover the following costs:

- Study specific screening tests and procedures
- Diagnostic, treatment and hospital costs from enrolment to 60 days or first discharge from hospital following enrolment, which ever is longer for all actively enrolled patients.
- Hospital cost for patients readmitted after first discharge for adverse events associated with TBM or study treatment during the 8 month TB treatment course.
- Study-related follow-up visits during the 8 month TB treatment course
- Treatment of any adverse events which are caused by study participation
- Travel expenses to attend follow-up visits for 8 months of treatment course (based on actual cost at standard OUCRU rates).

The study will not cover the cost of treating pre-existing diseases or those unrelated to study participation or the diagnosis and/or treatment of TBM.

### Participant Confidentiality

The trial staff will ensure that the participants’ anonymity is maintained. Participants will be identified only by initials and a participants ID number on the CRF, samples and any electronic database. All documents will be stored securely and only accessible by trial staff and authorised personnel.

## Sample Use and Storage

Samples collected will be used for the purpose of this study as stated in the protocol and stored for future use in studies not yet conceived within Viet Nam or abroad. Consent will be obtained from subjects for genetic testing and for sample storage and/or shipment of specific samples to collaborating institutions for investigations that cannot be performed locally. Any proposed plans to use samples other than for those investigations detailed in this protocol will be submitted to the relevant ethics committees prior to any testing.

The participants will be identified only by a study specific participant number and/or code in any database. The name and any other identifying detail will NOT be included in any study data electronic file.

# FINANCE AND INSURANCE

The conduct of this study is funded by the Wellcome Trust and sponsored by the University of Oxford. The University has a specialist insurance policy in place: - Newline Underwriting Management Ltd, at Lloyd’s of London – which would operate in the event of any participant suffering harm as a result of their involvement in the research.

# PUBLICATION POLICY

The primary outcome data will be analysed and reported in a publication. The authors (and their respective positions in the author list) will be agreed prior to the start of the study in accordance with the guidelines of the International Committee of Medical Journal Editors.

# REFERENCES

1. Thwaites GE, Tran TH. Tuberculous meningitis: many questions, too few answers. Lancet neurology. 2005; **4**(3): 160-70.

2. Thwaites GE, Chau TT, Stepniewska K, Phu NH, Chuong LV, Sinh DX, et al. Diagnosis of adult tuberculous meningitis by use of clinical and laboratory features. Lancet. 2002; **360**(9342): 1287-92.

3. Thwaites GE, Chau TT, Farrar JJ. Improving the bacteriological diagnosis of tuberculous meningitis. Journal of clinical microbiology. 2004; **42**(1): 378-9.

4. Thwaites GE, Caws M, Chau TT, Dung NT, Campbell JI, Phu NH, et al. Comparison of conventional bacteriology with nucleic acid amplification (amplified mycobacterium direct test) for diagnosis of tuberculous meningitis before and after inception of antituberculosis chemotherapy. Journal of clinical microbiology. 2004; **42**(3): 996-1002.

5. Thwaites GE, Nguyen DB, Nguyen HD, Hoang TQ, Do TT, Nguyen TC, et al. Dexamethasone for the treatment of tuberculous meningitis in adolescents and adults. The New England journal of medicine. 2004; **351**(17): 1741-51.

6. Heemskerk D, Day J, Chau TT, Dung NH, Yen NT, Bang ND, et al. Intensified treatment with high dose rifampicin and levofloxacin compared to standard treatment for adult patients with tuberculous meningitis (TBM-IT): protocol for a randomized controlled trial. Trials. 2011; **12**: 25.

7. Torok ME, Nguyen DB, Tran TH, Nguyen TB, Thwaites GE, Hoang TQ, et al. Dexamethasone and long-term outcome of tuberculous meningitis in Vietnamese adults and adolescents. PLoS ONE. 2011; **6**(12): e27821.

8. Lammie GA, Hewlett RH, Schoeman JF, Donald PR. Tuberculous cerebrovascular disease: a review. The Journal of infection. 2009; **59**(3): 156-66.

9. Hektoen L. The vascular changes in tuberculous meningitis especially tuberculous endarteritis. J Exp Med. 1896; **1**: 112-63.

10. Rich AR, McCordock HA. The pathogenesis of tuberculous meningitis. Bull John Hopkins Hosp. 1933; **52**: 5-37.

11. Doniach I. Changes in the meningeal vessels in acute and chronic (streptomycin-treated) tuberculous meningitis. The Journal of pathology and bacteriology. 1949; **61**(2): 253-9, 4 pl.

12. Poltera AA. Thrombogenic intracranial vasculitis in tuberculous meningitis. A 20 year "post mortem" survey. Acta neurologica Belgica. 1977; **77**(1): 12-24.

13. Botting RM. Vane's discovery of the mechanism of action of aspirin changed our understanding of its clinical pharmacology. Pharmacological reports : PR. 2010; **62**(3): 518-25.

14. Raju N, Sobieraj-Teague M, Hirsh J, O'Donnell M, Eikelboom J. Effect of aspirin on mortality in the primary prevention of cardiovascular disease. Am J Med. 2011; **124**(7): 621-9.

15. Spite M, Serhan CN. Novel lipid mediators promote resolution of acute inflammation: impact of aspirin and statins. Circulation research. 2010; **107**(10): 1170-84.

16. Misra UK, Kalita J, Nair PP. Role of aspirin in tuberculous meningitis: a randomized open label placebo controlled trial. J Neurol Sci. 2010; **293**(1-2): 12-7.

17. Schoeman JF, Janse van Rensburg A, Laubscher JA, Springer P. The role of aspirin in childhood tuberculous meningitis. Journal of child neurology. 2011; **26**(8): 956-62.

18. Tobin DM, Vary JC, Jr., Ray JP, Walsh GS, Dunstan SJ, Bang ND, et al. The lta4h locus modulates susceptibility to mycobacterial infection in zebrafish and humans. Cell. 2010; **140**(5): 717-30.

19. Tobin DM, Roca FJ, Oh SF, McFarland R, Vickery TW, Ray JP, et al. Host genotype-specific therapies can optimize the inflammatory response to mycobacterial infections. Cell. 2012; 148(3): 434-46.

20. TR Hawn, SJ Dunstan, GE Thwaites, CP Simmons, NT Thuong, NTN Lan, HT Quy, TTH Chau, S Rodrigues, M Janer, LP Zhao, TT Hien, JJ Farrar, A Aderem. A polymorphism in human TIRAP is associated with increased susceptibility to meningeal tuberculosis. Journal of Infectious Diseases. 2006; **194**(8):1127-34.

21. [Thuong NT, Hawn TR, Thwaites GE, Chau TT, Lan NT, Quy HT, Hieu NT, Aderem A, Hien TT, Farrar JJ, Dunstan SJ.](http://www.ncbi.nlm.nih.gov/sites/entrez?Db=pubmed&Cmd=ShowDetailView&TermToSearch=17554342&ordinalpos=1&itool=EntrezSystem2.PEntrez.Pubmed.Pubmed_ResultsPanel.Pubmed_RVDocSum) A polymorphism in human TLR2 is associated with increased susceptibility to tuberculous meningitis. Genes Immun. 2007 **8(5)**:422-8.

22 Mwandumba HC, Russell DG, Nyirenda MH, Anderson J, White SA, Molyneux ME, Squire SB. 2004. Mycobacterium tuberculosis resides in nonacidified vacuoles in endocytically competent alveolar macrophages from patients with tuberculosis and HIV infection. J Immunol **172**: 4592-8

23. Yates RM, Hermetter A, Taylor GA, Russell DG. 2007. Macrophage activation downregulates the degradative capacity of the phagosome. Traffic **8**: 241-50

24. Marais S, Thwaites G, Schoeman JF, Torok ME, Misra UK, Prasad K, et al. Tuberculous meningitis: a uniform case definition for use in clinical research. The Lancet infectious diseases. 2010; **10**(11): 803-12.

# APPENDIX A: STUDY FLOW CHART

# APPENDIX B: SCHEDULE OF PROCEDURES

| **Procedures** | **Visits** | | | | | | | |
| --- | --- | --- | --- | --- | --- | --- | --- | --- |
|  | **Screening** | **Baseline** | **Day 3 (+/- 2 days)** | **Day 7**  **(+/- 2 days)** | **Weekly until discharge** | **Day 30**  **(+/- 5 days)** | **Day 60**  **(+/- 5 days)** | **Day 240**  **(+/- 7 days)** |
| Eligibility assessment | **X** |  |  |  |  |  |  |  |
| Patient information sheet and informed consent | **X** |  |  |  |  |  |  |  |
| Randomisation |  | **X** |  |  |  |  |  |  |
| Clinical assessment**^(a)^** |  | **(X)** |  | **(X)** | **(X)** | **(X)** | **(X)** | **X** |
| Lumbar puncture**^(b)^** | **(X)** |  |  |  |  | **(X)** | **(X)** |  |
| EDTA blood (5mls)^(c)^ |  | **X** |  |  |  |  |  |  |
| Heparinised blood (20mls) ^(d)^ |  | **X** |  |  |  |  |  | **X** |
| PK blood (2ml) |  |  | **X – 5 times between day 1-14** | | |  |  |  |
| Full blood count | **(X)** | **X^(g)^** |  | **(X)** | **(X)** | **(X)** | **(X)** |  |
| Urea, Creatinine, electrolytes (5mls clotted blood) | **(X)** | **X^(g)^** |  | **(X)** | **(X)** | **(X)** | **(X)** |  |
| Liver function tests (AST, ALT, bilirubin) | **(X)** | **X^(g)^** |  | **(X)** | **(X)** | **(X)** | **(X)** |  |
| Stool microscopy for RBC |  |  |  | **x** | **x** | **X** | **X** |  |
| MRI brain^(e)^ |  | **X** |  |  |  |  | **X** | **X** |
| Adverse event assessments ^(f)^ |  |  |  | **X** | **X** | **X** | **X** | **X** |

*() indicate tests that will have already been performed as part of standard clinical care.*

1. Includes neurological examination and assessment of consciousness. Active surveillance for gastro-intestinal bleeding and other potential side effects of aspirin. Glasgow outcome and modified Rankin scores will be assessed on day 60 and 240.
2. Includes microscopy, culture, and drug susceptibility tests for *Mycobacterium tuberculosis*; Gene Xpert MTB/RIF test on all samples; and measurement of CSF leucocytes, protein, glucose, and cytokines (TNF-α, IL-1β, IL-8, IL-10, IFNγ) and eicosanoids (15-epi-Lipoxin, Lipoxin A4, LTB4, PGE2, TBXB2, PGD2). Up to 10 ml of CSF will be taken. Any residual volume of CSF remaining from these lumbar puncture will be stored for the purposes of this study
3. For determination of LTA4H genotype and other genetic polymorphisms possibly linked to susceptibility and outcome from TBM
4. For assessment of antimicrobial activity of peripheral blood monocyte/macrophages.
5. MRI brain can be +/- 5 days from randomisation and the date of follow-up.
6. Active surveillance for known adverse reactions to aspirin, including gastro-intestinal bleeding, renal dysfunction, and hepatitis. Cerebral bleeding will be assessed on MRI brain performed at baseline and day 60.
7. Only necessary if screening blood is >48 hours prior to baseline

# APPENDIX C. TBM DIAGNOSTIC CRITERIA

|  |  | | Diagnostic score |
| --- | --- | --- | --- |
| **Clinical criteria** | (Maximum category score=6) | | |
|  | Symptom duration of more than 5 days | | 4 |
|  | Systemic symptoms suggestive of tuberculosis (one or more of the following): weight loss (or poor weight gain in children)<comma> night sweats<comma> or persistent cough for more than 2 weeks | | 2 |
|  | History of recent (within past year) close contact with an individual with pulmonary tuberculosis or a positive TST or IGRA (only in children <10 years of age) | | 2 |
|  | Focal neurological deficit (excluding cranial nerve palsies) | | 1 |
|  | Cranial nerve palsy | | 1 |
|  | Altered consciousness | | 1 |
| **CSF criteria** | (Maximum category score=4) | | |
|  | Clear appearance | | 1 |
|  | Cells: 10-500 per μl | | 1 |
|  | Lymphocytic predominance (>50%) | | 1 |
|  | Protein concentration greater than 1 g/L | | 1 |
|  | CSF to plasma glucose ratio of less than 50% or an absolute CSF glucose concentration less than 2.2mmol/L | | 1 |
| **Cerebral imaging criteria** | (Maximum category score=6) | | |
|  | Hydrocephalus | | 1 |
|  | Basal meningeal enhancement | | 2 |
|  | Tuberculoma | | 2 |
|  | Infarct | | 1 |
|  | Pre-contrast basal hyperdensity | | 2 |
| **Evidence of tuberculosis elsewhere** | (Maximum category score=4) | | |
|  | Chest radiograph suggestive of active tuberculosis: signs of tuberculosis=2; miliary tuberculosis=4 | | 2 or 4 |
|  | CT/ MRI/ ultrasound evidence for tuberculosis outside the CNS | | 2 |
|  | AFB identified or *Mycobacterium tuberculosis* cultured from another source-ie, sputum, lumph node, gastric washing, urine, blood culture | | 4 |
|  | Positive commercial M tuberculosis NAAT from extra-neural specimen | | 4 |
| **Diagnostic criteria based on total score:**  Possible TBM: score 6-9 (if no brain imaging) or 6-11 (if brain imaging)  Probable TBM: score >9 (if no brain imaging) or >11 (if brain imaging)  Definite TBM: acid-fast bacilli seen in CSF or *M. tuberculosis* cultured or detected by commercial NAAT in CSF | |  |  |

# APPENDIX D. VIET NAM NATIONAL TUBERCULOSIS PROGRAMME TREATMENT GUIDELINES FOR TUBERCULOSIS MENINGITIS

See the Viet Nam National Tuberculosis Programme website or the Vietnamese language version of this protocol.
